# Identification of Host Factors for Rift Valley Fever Phlebovirus

**DOI:** 10.3390/v15112251

**Published:** 2023-11-13

**Authors:** Velmurugan Balaraman, Sabarish V. Indran, Yonghai Li, David A. Meekins, Laxmi U. M. R. Jakkula, Heidi Liu, Micheal P. Hays, Jayme A. Souza-Neto, Natasha N. Gaudreault, Philip R. Hardwidge, William C. Wilson, Friedemann Weber, Juergen A. Richt

**Affiliations:** 1 Center of Excellence for Emerging and Zoonotic Animal Diseases, Department of Diagnostic Medicine/Pathobiology, College of Veterinary Medicine, Kansas State University, 1800 Denison Ave, Manhattan, KS 66506, USA; 2 Foreign Arthropod-Borne Animal Diseases Research Unit, United States Department of Agriculture, National Bio and Agro-Defense Facility, Agricultural Research Service, 1980 Denison Ave, Manhattan, KS 66506, USA; 3 Institute for Virology, FB10—Veterinary Medicine, Justus-Liebig University, 35392 Giessen, Germany

**Keywords:** RVFV, host factor, *WDR7* gene, MP-12, A549 cells, LACV, bunyavirus, phlebovirus

## Abstract

Rift Valley fever phlebovirus (RVFV) is a zoonotic pathogen that causes Rift Valley fever (RVF) in livestock and humans. Currently, there is no licensed human vaccine or antiviral drug to control RVF. Although multiple species of animals and humans are vulnerable to RVFV infection, host factors affecting susceptibility are not well understood. To identify the host factors or genes essential for RVFV replication, we conducted CRISPR-Cas9 knockout screening in human A549 cells. We then validated the putative genes using siRNA-mediated knock-downs and CRISPR-Cas9-mediated knock-out studies. The role of a candidate gene in the virus replication cycle was assessed by measuring intracellular viral RNA accumulation, and the virus titers were analyzed using plaque assay or TCID_50_ assay. We identified approximately 900 genes with potential involvement in RVFV infection and replication. Further evaluation of the effect of six genes on viral replication using siRNA-mediated knock-downs revealed that silencing two genes (*WDR7* and *LRP1*) significantly impaired RVFV replication. For further analysis, we focused on the *WDR7* gene since the role of the *LRP1* gene in RVFV replication was previously described in detail. *WDR7* knockout A549 cell lines were generated and used to dissect the effect of *WRD7* on a bunyavirus, RVFV, and an orthobunyavirus, La Crosse encephalitis virus (LACV). We observed significant effects of *WDR7* knockout cells on both intracellular RVFV RNA levels and viral titers. At the intracellular RNA level, *WRD7* affected RVFV replication at a later phase of its replication cycle (24 h) when compared with the LACV replication, which was affected in an earlier replication phase (12 h). In summary, we identified *WDR7* as an essential host factor for the replication of two different viruses, RVFV and LACV, both of which belong to the *Bunyavirales* order. Future studies will investigate the mechanistic role through which *WDR7* facilitates phlebovirus replication.

## 1. Introduction

Rift Valley fever phlebovirus (RVFV) is a mosquito-borne, segmented RNA virus that belongs to the family *Phenuiviridae*, genus phlebovirus. RVFV was first isolated and characterized in the Rift Valley of Kenya in 1931 [1] and is the causative agent of Rift Valley fever (RVF). It is endemic throughout sub-Saharan Africa [2], the Arabian Peninsula (Saudi Arabia and Yemen), and Mayotte [3,4]. RVFV can be naturally transmitted to and cause disease in several animal species such as cattle, sheep, goats, and camels [5,6,7]. We have recently shown that white-tailed deer are highly susceptible to experimental infection with RVFV [8]. RVF in livestock is characterized by abortion storms in pregnant ewes and pregnant cattle and up to 100% mortality in newborn animals [5,6,7]. In humans, RVFV infection may be subclinical or causes mild flu-like symptoms and sometimes severe disease with hepatitis, retinitis, and encephalitis [9,10] with a small number of cases being lethal [11]. RVFV can infect and replicate in a multitude of cell lines (e.g., neurons, epithelial cells, etc.) from different animal species such as frogs, pigs, elk, mule deer, pronghorn, and reptiles, among others [12,13,14,15,16,17]; this highlights the potential for the virus to infect a wide variety of animals.

RVFV is mainly transmitted by infected mosquitoes (*Culex* and *Aedes*), through direct contact with infected animal secretions and exudates [18,19], or through aerosol exposure [20]. Currently, there are no FDA-approved therapeutic drugs or licensed vaccines available to control RVF in humans [20]. There is a real risk of the introduction of arboviruses such as RVFV to non-endemic countries, such as Europe, Asia, and North America [21], where competent vector mosquito species (e.g., *Culex* and *Aedes*) are present [18,22,23,24]. Therefore, RVFV poses a global threat to the health of livestock and humans, and to animal trade and commerce [24].

The successful development of antiviral therapies requires detailed knowledge of viral protein function or of host factors that support virus replication [25]. RVFV enters cells via receptor-mediated endocytosis and releases its nucleocapsid after fusion of the virus envelope with endosomal membranes. After the completion of replication, the viral particles assemble and bud from the Golgi apparatus [26]. Like many other RNA viruses, RVFV depends on various host factors to complete its replication cycle [27,28,29]. Several groups have conducted exploratory studies aimed at finding host factors or co-factors that might play a role in RVFV replication [27,29,30,31,32,33,34,35,36,37]. Notably, other researchers have shown that the low-density lipoprotein receptor-related protein 1 (LRP1) [29,37] and heparin sulfate [33] play essential roles in the cell entry of RVFV. Furthermore, the exogenous administration of the LRP1 inhibitor mRAPD3 protected mice from infection with a virulent strain of RVFV [29]. Devignot et al. (2023) reported that a *LRP1* gene knockout in Huh cells significantly affected intracellular RVFV RNA accumulation [37]. Bracci et al. (2022) found that UBR4 depletion affects RVFV production and virus titers in mammalian and mosquito cells [36]. Although these studies have identified host factors in mouse cells associated with RVFV replication, none of the host factors were able to completely abolish productive RVFV infection in gene-edited knockout cells. This indicates that RVFV interacts with a multitude of host factors to complete its replication cycle, exploiting multiple redundant cellular pathways. Our studies had the following aims: (1) to identify unique host factors that could significantly affect RVFV infection and replication; (2) to identify host factors that could be used as a potential drug target; and (3) to identify host factors that are conserved between different host species. To this end, a genome-wide CRISPR-Cas9 knockout (GeCKO) screening in human A549 cells infected with the RVFV MP-12 vaccine strain was performed in order to identify the host factors essential for RVFV infection and replication. We identified the WD repeat domain 7 (*WDR7*) gene as a critical host factor that plays a role in the late phase of RVFV replication. In addition, the *WDR7* gene also plays a role in the replication of an orthobunyavirus, the La Crosse encephalitis virus.

## 2. Materials and Methods

### 2.1. Cells

A549 cells (ATCC^®^ CCL-185™, American Type Culture Collection, Manassas, VA, USA) were cultured in an F-12 medium (ATCC, Manassas, VA, USA), supplemented with 10% fetal bovine serum (FBS, R&D Systems, Minneapolis, MN, USA) and 1% penicillin–streptomycin solution (ThermoFischer Scientific, Waltham, MA, USA). The Vero-MARU cell line is a clone of the Vero cells derived from African green monkey kidney cells and obtained from the Middle America Research Unit (Corozal, Panama). These cells are susceptible to RVFV infection, and they are the standard cell line used for all our plaque-based titration assays. The Vero-MARU, MRC-5 (ATCC^®^ CCL-171™), and Vero E6 (ATCC^®^ CRL-1586™) cell lines were cultured in Dulbecco’s modified Eagle’s medium (DMEM, Corning, New York, NY, USA), supplemented with 5% FBS (R&D Systems, USA) and 1% penicillin–streptomycin solution (ThermoFischer Scientific, USA). All mammalian cells were maintained at 37 °C under a 5% CO_2_ atmosphere. The *Aedes albopictus* larva (C6/36, ATCC^®^ CRL-1660™) cells were maintained at 28 °C and cultured in an L-15 medium (ATCC, USA), supplemented with 10% insect cell culture tested FBS (IFBS, Catalog Number: F4135, Sigma-Aldrich, St. Louis, MO, USA), 10% tryptose phosphate broth (TPB, Catalog Number: T9157, Sigma-Aldrich, USA), and 1% penicillin–streptomycin solution (ThermoFischer Scientific, USA).

### 2.2. Virus Strains

The RVFV MP-12 vaccine strain provided by the US Army Medical Research Institute for Infectious Diseases (Fort Detrick, MD, USA) [38] was propagated in MRC-5 cells; the RVFV Kenya 128B-15 virulent strain was provided by R. Bowen, Colorado State University (Fort Collins, CO, USA), with authorization from B. Miller, Centers for Disease Control, Fort Collins, CO, USA [39] and was grown in C6/36 cells. La Crosse Encephalitis virus (LACV), NR-540, was obtained from BEI Resources, NIAID, and propagated in Vero E6 cells. RVFV MP-12 and Kenya 128B-15 strains were titered using plaque assay and the LACV by TCID_50_-CPE assay. We used the attenuated MP-12 vaccine strain, which is classified as a BSL-2 agent, to perform our initial studies, including the pooled CRISPR-KO screening, as well as the siRNA gene knock-down and CRISPR-Cas9 knock-out experiments. Subsequently, we conducted studies with the virulent Kenya 128B-15 strain, which is a wild-type RVFV strain, in a BSL-3+ facility at the Biosecurity Research Institute of Kansas State University (Manhattan, KS, USA) to confirm the critical role of WDR7 in RVFV infections.

### 2.3. Generation of GeCKO-A549 Cell Line and RVFV Screening

**Figure 1:**
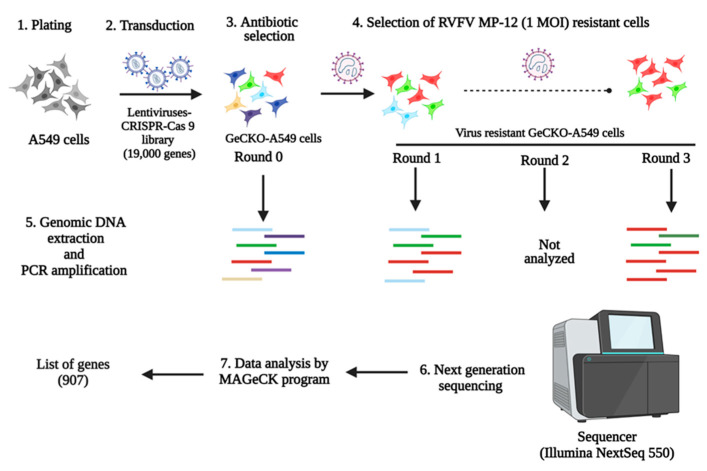
Schematics of GeCKO-A549 cells generation, selection, NGS, and data analysis. A549 cells were transduced with the lentivirus-CRISPR-Cas9 library to generate GeCKO-A549 cells. Then, the GeCKO-A549 cells were subjected to three rounds of infection with the RVFV MP-12 (1 MOI) virus. The genomic DNA of round 0 GeCKO-A549 cells, round 1, and round 3 GeCKO-A549cells were sequenced using the Illumina NextSeq 550 platform. The output NGS data were analyzed using the MaGeCK program to generate the list of genes involved in RVFV replication.

The lentiCRISPRv2 library, which targets 19,000 human genes, was obtained from Addgene (Catalog Number: 1000000048, Addgene, Watertown, MA, USA). The library contains non-target control sgRNAs, sgRNAs targeting miRNAs, and six unique sgRNAs designed to target each individual human gene. To generate GeCKO-A549 cells, a pooled lentivirus library was created using the lentiCRISPRv2 plasmids, following previously described methods [40,41]. A puromycin (Catalog Number: A1113803, Sigma-Aldrich, St. Louis, MO, USA) cytotoxicity curve was performed on A549 cells, and the puromycin concentration used was determined to be 2 μg/mL medium. Then, the transduction efficiency of the lentivirus library on A549 cells was determined as previously described [40,41]. Two independently pooled GeCKO-A549 cell lines were generated and subjected to forward genetic screening. Briefly, 80 million GeCKO-A549 cells were subjected to up to three rounds of cytolytic infection with RVFV MP-12 (1 MOI), and the surviving cells were expanded between each round of infection. The gDNAs were extracted from the round 0 (mock-infected), round 1, and 3 virus infections of GeCKO-A549 cells using a MIDI gDNA Extraction Kit (Qiagen, Germantown, MD, USA). The sgRNA’s DNA copies were PCR-amplified from the extracted gDNAs for next-generation sequencing (Figure 1). Next-generation sequencing was performed using NextSeq (Illumina, San Diego, CA, USA), and the obtained data were analyzed using MAGeCK software version 0.5.9. The ranking of genes was determined using robust ranking aggregation [42].

### 2.4. siRNA Transfection

Six genes were selected after NGS analysis of the RVFV-resistant GeCKO-A549 cells for siRNA gene knockdown studies (Table S1). The gene targets for the siRNAs were as follows: siRNAs: NTC-non-target control (Catalog Number: D-001206-14-05); WDR7 (Catalog Number: M-012867-01-0005); LRP1 (Catalog Number: M-004721-01-0005); EXOC4 (Catalog Number: M-013068-01-0005); SLC35B2 (Catalog Number: M-007543-01-0005); CT47A1 (Catalog Number: M-184305-00-0005); and EMC3 (Catalog Number: M-010715-00-0005); they were commercially purchased (Dharmacon, Lafayette, CO, USA). The positive control siRNA-si46N [43] targeting the RVFV nucleoprotein was obtained from Integrated DNA Technologies (Coralville, IA, USA). A549 cells were plated in 96-well plates and incubated overnight. The cells were transfected with siRNAs (50 nM) using lipofectamine RNAimax reagent (ThermoFischer Scientific, USA). Forty-eight hours post-transfection, cells were infected with RVFV MP-12 at 0.1 MOI, and the infected cell supernatant was collected at 24 h post-infection (h pi). The virus titer of the supernatants was determined using plaque assay on Vero-MARU cells.

### 2.5. RT-qPCR for Host Gene Expression

To confirm gene knockdown, two-step RT-qPCR assays were performed. Briefly, A549 cells were transfected with gene-specific siRNAs at 50 nM, and 48 h later, the total cellular RNA was extracted. The RNA extraction was performed using the RNAqueous Micro Total RNA Isolation Kit (ThermoFischer Scientific, USA) following the manufacturer’s protocol. Prior to cDNA synthesis, residual gDNA was removed from the extracted RNA using the DNAse I enzyme (ThermoFischer Scientific, USA). Then, 400 ng of RNA was used for cDNA synthesis using the Superscript IV First-Strand Synthesis Kit with oligo dT primers (ThermoFischer Scientific, USA) following the manufacturer’s protocol. All RT-qPCR reactions were performed in a CFX96 Real-Time thermocycler (BioRad, Hercules, CA, USA). The standard real-time qPCR assays were performed using Perfecta Fastmix II (Quanta BioSciences, Beverly, MA, USA) with gene-specific primers (Table S2). The glyceraldehyde 3-phosphate dehydrogenase (*GAPDH*) gene was used as an internal control [44]. The percentage gene knockdown was calculated using the 2^–∆∆^CT method [45].

### 2.6. Generation of WDR7 Knock-Out (KO) Cells

Two WDR7 knockout (KO) cell lines and a control non-KO cell line were generated as previously described [41]. *WDR7*-gene targeting sgRNAs (sgRNA 1: 5′GTGACATCCTGTTACGATCG3′ and sgRNA 5: 5′AAGATGGCAAGATCGATGCT’3) were applied to generate two WDR7 KO cell lines: the WDR7 KO cell line 1 (WDR7 KO 1) and 2 (WDR7 KO 2). The non-KO control cell line (CT) was generated via the transduction of the lentiCRISPRv2 vector with the Cas9 backbone without sgRNAs. LentiCRISPRv2 plasmids 1 and 5 containing sgRNAs specific for the *WDR7* gene were purchased from Genescript, USA. The control and WDR7 sgRNA plasmids were packaged into lentivirus, and the A549 cells were transduced with 0.5 MOI of lentivirus. The transduced cells were kept under puromycin selection and passed three times prior to testing. The gDNA of the two WRD7 KO cell lines were extracted using the DNAeasy kit (Qiagen, Germantown, MD, USA), and the gDNA was PCR-amplified for NGS analysis. Sequencing was performed using a MiSeq (Illumina, USA). The indel percentage of the KO cell lines was calculated using a Python script [41].

### 2.7. Western Blot Analysis

A549 cells, CT cells, and WDR7 KO 1 and 2 cells at passage 3 were used for Western blot analyses. The cell lysates were prepared as previously described [46]. Cell lysates containing 55.0 µg total protein were loaded onto 4–12% Bis-Tris polyacrylamide gels (ThermoFischer Scientific, USA) and transferred onto a polyvinylidene difluoride (PVDF) membrane using a Trans-Blot Turbo Transfer Pack (BioRad, USA). The membrane was blocked using 5% skim milk and then incubated with a primary polyclonal antibody against WDR7 (diluted 1:500, Catalog Number: sab2109026, Sigma-Aldrich, USA) or β-actin (diluted 1:5000, Catalog Number: ab20272, Abcam, Waltham, MA, USA) for 1 h at room temperature. The membrane was then incubated with horseradish peroxidase (HRP)-conjugated polyclonal goat anti-rabbit immunoglobulin (diluted 1:1000, Catalog Number: 31460, ThermoFischer Scientific, USA). The target proteins were detected using Super Signal West Femto Maximum Sensitivity Substrate according to the manufacturer’s protocol (Catalog Number: 34095, ThermoFischer Scientific, USA). The images were taken using a ChemiDoc MP Imaging System (BioRad, USA).

### 2.8. Testing of WDR7 KO Cells for Virus Replication 

The non-knockout control (CT) and WDR7 KO cell lines were seeded onto 96-well plates and allowed to incubate overnight. Afterward, the cells were infected with either RVFV MP-12, RVFV Kenya 128B-15, or La Crosse encephalitis virus (LACV) at 0.1 MOI, and the cell supernatants were collected at 6, 12, 24, or 48 h pi. The titer of collected supernatants was determined using the plaque assay (RVFV) or the TCID_50_-CPE (LACV) assay.

### 2.9. Intracellular Viral RNA Accumulation Assay

The viral RNA accumulation was determined at various time points (0, 2, 5, and 24h pi) as previously described [37,47]. The CT and WDR7 KO 1 cells were plated in 6-well plates. Twenty-four hours later, cells were infected with RVFV MP-12 or LACV at a MOI of 0.1 for 1 h at 0 °C to allow for virus attachment and entry. For the 0 h infection, immediately after infection, the cells were washed three times with 1× phosphate-buffered saline (PBS (pH = 7.2–7.6), Catalog Number: P4417, Sigma-Aldrich, St. Louis, MO, USA), lysed in 350 μL RLT buffer (Qiagen, Germantown, MD, USA), and then stored at −80 °C for subsequent use. For the post-infection time points, the cells were washed once with 1× PBS after the initial 1 h of incubation and then incubated with 2 mL of pre-warmed fresh medium. At 2 h pi, cells were first trypsinized and collected into microcentrifuge tubes. Then, the trypsinized cells were washed three times with 1× PBS via centrifugation at 10,000× *g* for 5 min. The cell pellets were lysed in RLT buffer and stored at −80 °C till further use. For the 5 and 24 h time points, the cells were washed once with 1× PBS and lysed in RLT buffer for 10 min prior to storage at −80 °C for future analysis. The total cellular RNA was extracted using an RNeasy Mini Kit (Qiagen, Germantown, MD, USA). One-step RT-qPCR assays were performed using q-script XLT (2×) Master mix (Quanta BioSciences, Beverly, MA, USA) with virus gene-specific primers and probes (Table S3); the phosphoglycerate kinase (*PGK1*) gene was used as an internal housekeeping control gene [44]. Respective gene expressions were calculated using the 2^–∆∆^CT method [48].

### 2.10. Plaque Assay

Vero-MARU cells were seeded in 12- or 24-well plates and incubated at 37 °C and 5% CO_2_ overnight. After overnight incubation, the cells were infected with RVFV for one hour, and then the medium was replaced with an overlay of 1% methylcellulose-1× MEM (ThermoFischer Scientific, USA), 5% FBS, 1% antibiotics/antimycotic. The cells were incubated for 5–7 days and then stained and fixed with a 5% crystal violet fixative solution. The plaques were counted, and the titer was expressed as pfu/mL.

### 2.11. TCID_50_-CPE Assay

Vero E6 cells were seeded in 96-well plates one day prior to infection. Ten-fold serial dilutions of LACV were prepared in 96-well plates in DMEM supplemented with 5%FBS and 1% antibiotic–antimycotic solution. The diluted viral suspensions were then added to Vero E6 cells. Three to four days after infection, the cells were visually observed under a microscope for CPE, and the titer was calculated using the Spearman–Karber method [49].

### 2.12. Statistical Analysis

The statistical analyses performed in this study are described in the figure legends. All the statistical tests were carried out using GraphPad Prism version 9.3.0.3.

## 3. Results

### 3.1. Identification of Host Factors Involved in RVFV Replication 

To identify the genes potentially involved in RVFV replication, we performed CRISPR-Cas9 knockout screening in A549 cells. The A549 type II alveolar human cell line was selected for the screening because it is susceptible to RVFV and can be easily transduced with the human GeCKO library. The GeCKO-A549 cells were subjected to three rounds of infection with the RVFV MP-12 vaccine strain to select for resistance to RVFV infection and to identify the key host factors that are required for virus replication. Extensive cytopathic effect (CPE) was observed during the first round of infection. Surviving cells were reinfected, and the CPE was much less extensive during the second and third rounds of infection. To assess the susceptibility of GeCKO-A549 cells after three rounds of RVFV infection, virus growth kinetic assays were performed. A significant difference in MP-12 virus titers was observed between round 0 and round 3 GeCKO-A549 cells at 24 and 48 h post-infection (h pi) (Supplementary Figure S1), indicating that the round 3 GeCKO-A549 cells were less permissive to RVFV infection. Next, the putative genes involved in RVFV replication were determined by analyzing the NGS data from round 0, round 1, and round 3 GeCKO-A549 cells. Our analysis of round 3 GeCKO-A549 cells revealed that 907 genes (*p*-value < 0.05) seem to be involved in RVFV MP-12 replication (Supplementary File S1). For further analysis, we selected the six top genes significantly enriched in round 3 GeCKO-A549 cells: *LRP1*, *SLC35B2*, *EMC3*, *WDR7*, *EXOC4*, and *CT47A1* (Supplementary File S1). We did not investigate the other top two genes, *ART3* and *CEBPD* (Supplementary File S1), as they were associated with essential cellular functions.

### 3.2. Validation of Genes from the Pooled GeCKO-A549 Cell Screening

**Figure 2:**
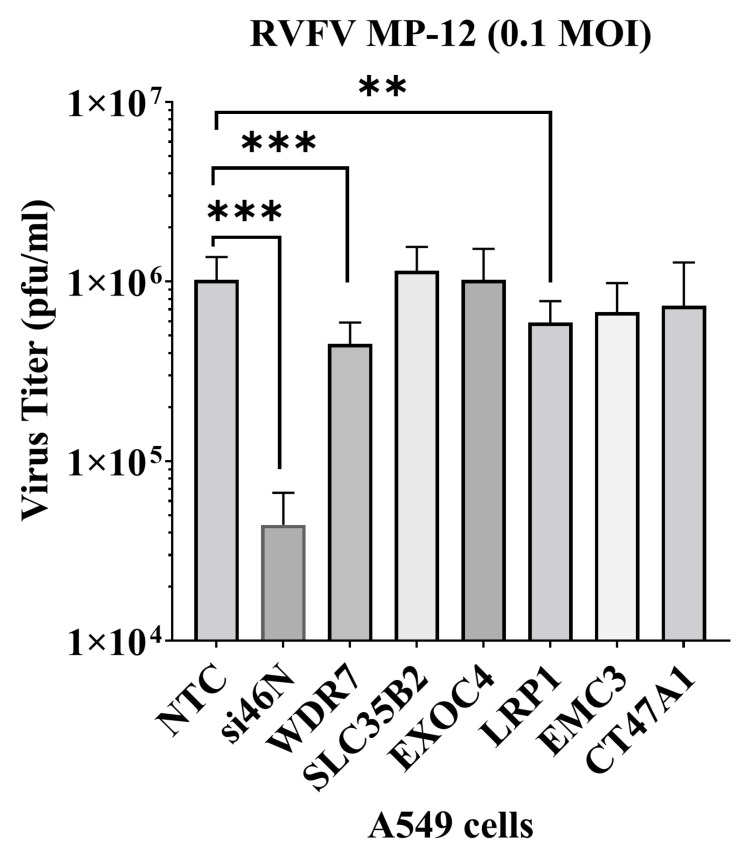
Validation of gene hits via siRNA gene knockdown study. A549 cells were transfected with 50 nM of siRNAs. At 48 h post-transfection, the cells were infected with RVFV MP-12 virus at 0.1 MOI. At 24 h post-infection, the supernatant was collected and titered using plaque assay. NTC-non-target control siRNA; si46N-anti-RVFV siRNA; and *WDR7*, *SLC35B2*, *EXOC4*, *LRP1*, *EMC3*, *CT47A1* gene-specific siRNAs were transfected. Each bar represents the average virus titer (pfu/mL) along with the corresponding standard deviation. Statistical analysis was performed on two independent experiments with four replicates for each, using the Mann–Whitney U test and independent Student’s *t*-test (** *p*-value < 0.005, *** *p*-value < 0.001).

To assess the effect of the six top genes enriched in round 3 GeCKO-A549 cells on RVFV replication, we used siRNA-mediated gene silencing (gene knockdown) in A549 cells. Gene knockdown was confirmed via respective RT-qPCR assays and the average reduction in gene expression ranged from approximately 55% to 90% (Supplementary Figure S2). After gene knockdown, the cells were infected with RVFV MP-12 virus at 0.1 MOI for 24 h, supernatants were harvested, and extracellular virus titer was determined using plaque assay. There was an average of 56% or 42% reduction in virus titer upon *WDR7* and *LRP1* knockdown, respectively, compared with non-target control (NTC) siRNA targeting the firefly luciferase mRNA (Figure 2). The positive control siRNA, siRNA-si46N, targeted the N protein gene of RVFV and caused a reduction of approximately 96% in virus titer compared with the negative control group. We observed no significant effect on virus titers following the knockdown of the other four selected top genes, namely *EXOC4*, *CT47A1*, *EMC3*, and *SLC35B2* (Figure 2). These results demonstrate that the knockdown of *WDR7* and *LRP1* significantly impaired RVFV replication. Given that the role of the *LPR1* gene in RVFV replication has been recently demonstrated [29,37], we focused our further analysis on the newly discovered putative RVFV host factor *WDR7*.

### 3.3. Generation and Characterization of Knockout Cells

**Figure 3:**
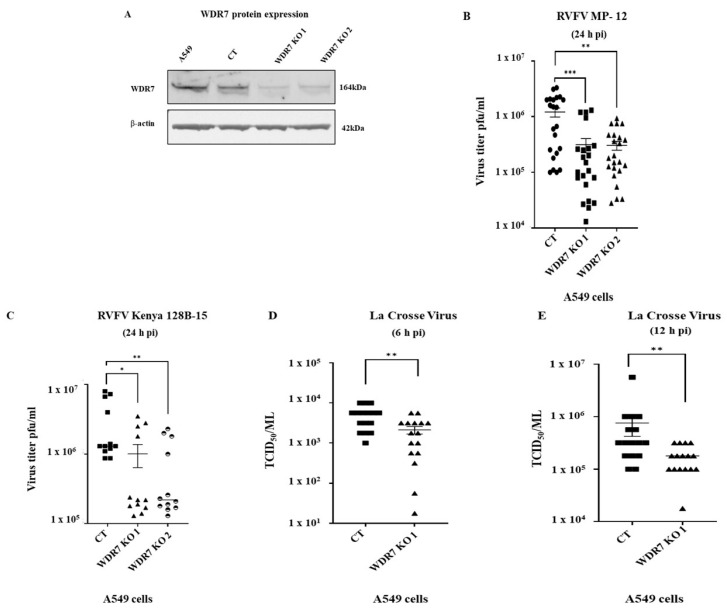
Effect of *WDR7* gene knockout (KO) on virus production of bunyaviruses: (**A**) A549 cells CT (non-knockout control) cells and *WDR7* gene KO cell lines 1 and 2 were analyzed for WDR7 protein expression via Western blot using a WDR7-specific polyclonal antibody. (**B**–**E**) CT cells and WDR7 KO A549 cells were infected with RVFV MP-12 vaccine strain (**B**), with the wild-type RVFV Kenya 128B-15 strain (**C**), or with La Crosse encephalitis virus (**D**,**E**) at 0.1 MOI. The supernatant was collected at 6, 12 or 24 h post-infection (h pi) and titered using plaque assay (RVFV) or TCID_50_-CPE assay (LACV). RVFV MP-12 testing on A549 CT cells and WDR7 KO lines 1 or 2, involved three to five independent experiments with three to four technical replicates each. RVFV Kenya 128B-15 testing involved independent experiments with three technical replicates each. LACV testing was performed in two independent experiments with eight technical replicates each. Statistical analysis was performed using the Mann–Whitney U test and independent Student’s *t*-test (* *p*-value < 0.05, ** *p*-value < 0.005, *** *p*-value < 0.001).

To investigate the role of *WRD7* in the RVFV replication cycle, we employed highly enriched sgRNAs targeting the *WDR7* gene to generate two knockout (KO) A 549 cell lines: WDR7 KO line 1 and WDR7 KO line 2. The established WRD7 knockout cells were analyzed via NGS sequencing, which confirmed indels in nearly 100% of the WDR7 KO cells (99% and 98%, respectively, for the two WDR7 KO cell lines 1 and 2, (Supplementary Table S1)). There was also a significant decrease in WDR7 protein expression in the WDR7 KO cell lines as compared to the control and non-transduced A549 cells (Figure 3A). However, we noted the presence of a faint WDR7 band in both WDR7 KO cells.

To ensure the authenticity of the A549 control cells, we sequenced the *WDR7* gene at the target site and found that the *WDR7* gene was not mutated in the CT cells (Supplementary File S2); also, the WDR7 protein expression in CT cells was at a similar level to the level in the non-transduced A549 cells (Figure 3A). Moreover, the CT cells showed comparable levels of virus replication to the non-transduced wild-type A549 cells (Supplementary Figure S3E). Additionally, cell viability did not differ significantly between the WDR7 KO cell lines 1 and 2, and the CT cell line, neither prior to nor after RVFV MP-12 infection (Supplementary Figure S3A,D).

### 3.4. Effect of WDR7 Gene Knockout on RVFV and LACV Infection

Next, we infected the two WDR7 KO cell lines with the RVFV MP-12 strain at 0.1 MOI and determined the extracellular virus titers using plaque assay. The *WRD7* gene KO resulted in a significant reduction of approximately 74% in virus titer compared with CT cells at 24 h post-infection (Figure 3B), while no difference in virus titers was observed at 48 h post-infection (Supplementary Figure S4A). We then evaluated the effect of the *WDR7* gene KO on the virulent RVFV strain Kenya 128B-15. Our results showed an average reduction of 66% and 75% in RVFV Kenya 128B-15 titers using WDR7 KO cell lines 1 and 2, respectively, when compared to the CT cells (Figure 3C). Taken together, these findings support the results obtained using the *WDR7* siRNA knockdown experiments, and confirm the critical role of the *WDR7* gene in the RVFV replication cycle. 

In addition, we evaluated if the *WDR7* gene plays a role in the replication cycle of an orthobunyavirus. For this purpose, we used the La Crosse encephalitis virus (LACV) and infected the CT cells and WDR7 KO cell 1 line with the LACV; the cell supernatant was collected at various time points post-infection, and the virus titer was determined using TCID_50_-CPE assay. The results showed an average reduction in LACV titer of 57% and 77% at 6 h pi and 12 h pi, respectively, in the WDR7 KO 1 cell line compared with the control CT cells (Figure 3D,E). At 24 h pi, the reduction in virus titer was approximately 39% which was statistically not significant (Supplementary Figure S4B). Overall, these findings highlight the importance of the *WDR7* gene in LACV replication.

### 3.5. WDR7 Gene Knock-Out Impairs RVFV and LACV Intracellular RNA Accumulation

**Figure 4:**
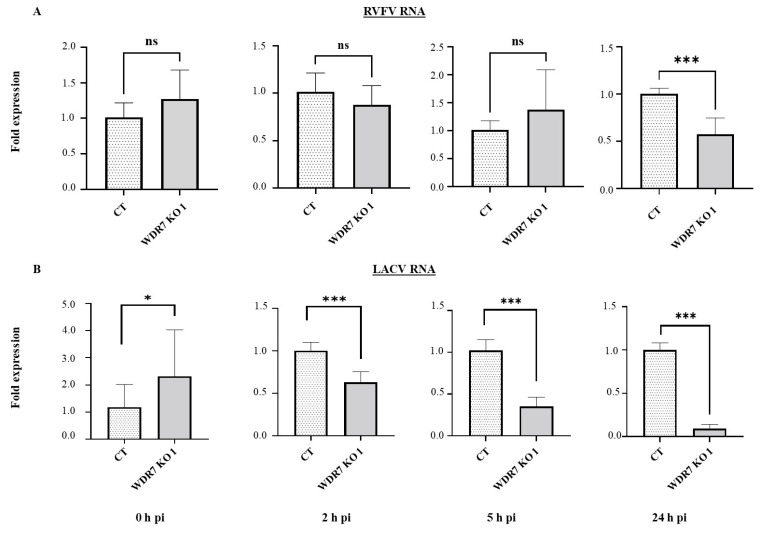
Viral RNA accumulation at various time points post-infection in WDR7 knockout (KO) cells. CT and WDR7 KO 1 cells were infected with (**A**) RVFV MP-12 vaccine strain or (**B**) LACV, both at 0.1 MOI. Total cellular RNA was harvested at various hours post-infection (h pi). One-step RT-qPCR was performed to detect the level of viral RNA using the *PGK1* gene as an internal control. CT and WDR7 KO 1 cells were utilized. Each bar graph represents the average fold change in viral RNA expression, along with the corresponding standard deviation. Statistical analysis was performed on three independent experiments with two to three technical replicates for each, using the Mann–Whitney U test and independent Student’s *t*-test (* *p*-value < 0.05, *** *p*-value < 0.001, ns, non-significant).

To investigate the role of WDR7 in the RVFV and LACV replication cycle, we quantified intracellular viral RNA accumulation at 0 h pi (attachment phase), 2 h pi (entry phase), 5 h pi (replication phase), and 24 h pi (late phase of replication) using previously established protocols [37,47]. At 0, 2, and 5 h pi, there was no significant difference in RVFV RNA accumulation between the CT control cells and the WDR7 KO cell line 1 (Figure 4A). However, at 24 h pi, we observed a significant reduction in virus RNA accumulation between the WDR7 KO and CT control cells (Figure 4A). When we infected the WDR7 KO cell line 1 and CT control cells with the LACV, we found that the WDR7 KO cells had higher levels of LACV RNA accumulation at 0 h pi, i.e. in the attachment phase, compared with the CT control cells (Figure 4B). However, at early time points, 2 and 5 h pi, and up to 24 h pi, we observed a significant reduction in LACV RNA accumulation in WDR7 KO 1 cells when compared to the CT control cells (Figure 4B). These results suggest that *WDR7* gene disruption affects intracellular viral RNA accumulation primarily at the late phase of the RVFV replication cycle and at the early phase of the LACV replication cycle.

## 4. Discussion

RVFV has a broad cell tropism and is reported to infect several animal and mosquito species. RVFV interacts with different host factors in a variety of cell types [29,30,31,32,33,34,36,37]. We identified WDR7, a member of the WD repeat protein family, as a host factor important in the lifecycle of RVFV and LACV bunyaviruses. We confirmed *WDR7* gene knock-out through NGS and indel analysis; Western blotting detected a faint band corresponding to the WDR7 protein in WDR7 knock-out cells. This minimal expression could be due to a single guide sgRNA inducing minor double-stranded DNA breaks that resulted in the production of some non-functional protein. Importantly, we demonstrated that the disruption of the *WDR7* gene impairs viral RNA accumulation and infectious virus production of two bunyaviruses, RVFV and LACV. However, the exact role of WDR7 in the replication cycle of these viruses needs further investigation. Previous studies have shown that WDR7 also plays a significant role in the replication cycle of other RNA viruses such as the dengue virus, Zika virus, West Nile virus [50], and influenza A virus [51].

WDR7 has been associated with V-ATPase, which mediates intracellular vesicle acidification in mouse kidney cells [52], suggesting that WDR7 could play a role in endocytosis or secretory pathways within the virus replication cycle. Here, we demonstrated that WDR7 affects the late phase of the RVFV replication cycle, as shown by the reduction in intracellular viral RNA in WDR7 KO cells compared with non-KO CT cells at 24 h pi. Combined with the significant lower levels of infectious RVFV in WDR7 KO cell supernatants compared with CT control cells, this might suggest that WDR7 impacts virus egress and release. In contrast, for the LACV, WDR7 seems to affect early stages of virus replication, since a significant reduction in both intracellular viral RNA and infectious virus production was found at an early time point post-infectionin WDR7 KO cells compared with CT control cells. This could be due to the fact that the WDR7 gene KO might affect the expression or function of other host factors involved in virus attachment and/or virus entry, or that the *WDR7* gene knock-out affects the conformation or expression of cell surface molecules needed for attachment and/or entry. While both RVFV and LACV belong to the order *Bunyavirales*, they belong to different genera: RVFV is a phlebovirus and LACV is a orthobunyavirus. This could be one explanation for the WDR7 effect at different replication stages for RVFV and LACV.

Interestingly, the effect of the knock-out of the *WDR7* gene in A549 cells on virus replication appeared to diminish at later replication time points for both RVFV and LACV. This pattern is consistent with the findings reported by Bracci et al. (2022), who observed a similar trend in RVFV replication in UBR4 knockout cells, with a significant reduction at 24 h pi but no significant effect at 48 h pi [36]. This suggests that the RVFV and LACV have the ability to utilize multiple alternative host factors and pathways to complete their replication cycle. We also observed a significant reduction in the LACV viral RNA at later time points but not in infectious virus production. This result could be attributed to various factors such as a gene knock-out effect on late RNA synthesis, increased RNA degradation, or decreased RNA stability in the absence of WDR7; all these could affect viral RNA synthesis or RNA stability, while virus release or egress was unaffected.

Overall, this study highlights the importance of the *WDR7* gene in the replication of two different viruses from the *Bunyavirales* order: RVFV phlebovirus and LACV orthobunyavirus. It also suggests that WDR7 could be a potential target for the development of antiviral therapies. Further research, including in vivo studies including *WDR7* gene knock-out mouse models, is needed to fully elucidate the role of WDR7 in bunyavirus replication.

## Data Availability

Data can be obtained from the authors upon request.

## Supplementary Materials

The following supporting information can be downloaded at: https://www.mdpi.com/article/10.3390/v15112251/s1, Figure S1: RVFV growth kinetics on wild-type and round 3 GeCKO-A549 cells; Figure S2: Confirmation of WDR7 gene knock-down; Figure S3: Cell viability and virus titers of wild-type, CT, and WDR7 gene knock-out A549 cell lines; Figure S4: Effect of WDR7 gene KO cell lines 1 and 2 on virus replication of RVFV and LACV at 48 or 24 hpi, respectively. Table S1: Location of the indels in the WDR7 gene of knock-out A549 cells; Table S2: Primers used for RT-qPCR, NGS and Sanger sequencing; Table S3: Primer and probes for the detection of viral RNA by RT-qPCR; Supplementary File S1: List of genes enriched in round 1 and round 3 virus-infected GeCKO A549 cells; Supplementary File S2: I. Predicted WDR7 gene sequences of wild-type and WDR7 knock-out A549 cells; II. WDR7 gene sequences of wild-type and WDR7 knock-out A549 cells obtained by Sanger sequencing.
