# Supplementary material for: Identification of Host Factors for Rift Valley Fever Phlebovirus
Source: Viruses. 2023 Nov 13;15(11):2251. doi: 10.3390/v15112251 (PMC10675714; doi:10.3390/v15112251)
Supplement: Supplementary file 1 [file viruses-15-02251-s001.zip › Supplementary File S2 corrected JAR.pdf]

Supplementary File S2:
I – Predicted *WDR7* gene sequences
II - Sanger sequencing

I. Predicted *WDR7* gene sequences of wild-type and WDR7 knock-out A549 cells. A, B. Wild-type A549 cells. **C-F.** WDR7 knock-out cell line 1. **G-J.** WDR7 knock-out cell line 2. The sgRNA binding region of exon 2 and exon 17 is highlighted in yellow. The *indels* and mutations are highlighted in red.

A) Full nucleotide sequence of *WDR7* gene exon 1-28 in wild-type A549 cells:
ATGGCAGGAAACAGCCTTGTTCTACCCATTGTTCTTTGGGGTCGAAAAGCGCCCACACAT
TGCATCTCAGCCGTACTTTTAACAGATGATGGGGCCACGATCGTAACAGGATGTCACGAC
GGACAAATATGTCTCTGGGATCTTTCAGTAGAACTGCAAATTAATCCTCGAGCACTGTTG
TTTGGTCATACAGCATCAATCACTTGTTTGTCTAAAGCTTGTGCTTCCAGTGACAAACAG
TATATTGTGAGTGCATCTGAAAGTGGAGAGATGTGCCTCTGGGATGTGAGTGATGGCAGA
TGTATTGAATTTACAAAATTAGCTTGCACACATACTGGCATACAGTTCTACCAGTTCTCT
GTTGGGAATCAGCGAGAAGGAAGGCTTTTATGCCACGGACATTACCCTGAAATCCTTGTT
GTGGATGCTACCAGCCTTGAAGTATTATACTCCTTAGTATCAAAGATATCACCAGACTGG
ATTAGCTCCATGAGTATTATTCGATCCCACCGAACACAAGAGGACACAGTGGTAGCACTC
TCGGTGACTGGCATCCTGAAGGTCTGGATTGTTACCTCGGAAATAAGTGACATGCAGGAT
ACTGAGCCAATATTTGAGGAGGAATCCAAACCAATTTATTGTCAGAATTGCCAAAGCATC
TCTTTTTGTGCATTTACACAAAGGTCACTTTTGGTTGTGTGTTCCAAATATTGGAGGGTG
TTCGATGCCGGAGACTATTCCTTGTTGTGTTCAGGTCCTAGTGAAAATGGACAGACATGG
ACCGGGGGGGACTTTGTCTCATCAGATAAAGTCATCATTTGGACAGAAAATGGGCAAAGT
TATATTTACAAACTACCTGCCAGTTGCCTTCCAGCTAGTGATTCATTCCGCAGTGATGTG
GGGAAGGCAGTTGAAAATTTAATTCCTCCTGTACAACATATCCTCTTGGATCGAAAAGAT
AAAGAGTTGCTAATTTGTCCTCCTGTTACTCGGTTCTTCTATGGATGCAGAGAATATTTC
CATAAACTGTTAATTCAGGGTGATTCTTCTGGAAGGTTGAATATTTGGAACATATCAGAC
ACAGCTGATAAACAGGGAAGTGAAGAAGGGCTGGCAATGACAACTTCTATTAGTTTGCAA
GAGGCATTTGATAAACTGAATCCTTGTCCTGCTGGAATTATAGATCAGCTGAGTGTGATT
CCCAATAGTAATGAACCTCTTAAAGTAACTGCAAGTGTGTACATACCAGCACATGGACGA
CTTGTTTGTGGTCGTGAAGATGGAAGCATAGTTATTGTACCTGCCACACAGACGGCCATA
GTACAGCTGTTGCAAGGGGAACACATGCTCAGAAGAGGTTGGCCACCTCACAGAACACTC
CGTGGTCATCGGAACAAAGTCACATGTTTGCTATATCCTCATCAGGTCTCAGCTCGGTAT
GATCAAAGATACCTGATATCTGGAGGTGTGGATTTTTCAGTCATAATTTGGGACATATTT
TCTGGAGAAATGAAACATATCTTCTGTGTTCATGGTGGTGAGATTACTCAACTTCTAGTT
CCACCTGAAAACTGTAGTGCAAGAGTACAGCACTGCATCTGCTCTGTAGCCAGTGACCAC
TCAGTAGGACTTCTAAGTTTGCGAGAGAAAAAATGCATAATGTTGGCATCTCGTCACCTT
TTTCCTATTCAAGTAATCAAATGGAGGCCTTCTGATGATTACCTGGTGGTGGGGTGTTCA
GATGGTTCTGTGTACGTCTGGCAAATGGATACTGGTGCATTGGATCGTTGTGTGATGGGG
ATAACAGCAGTTGAGATTCTAAACGCTTGTGATGAAGCTGTTCCTGCTGCTGTTGATTCA
CTTAGTCATCCAGCAGTCAACCTAAAACAAGCTATGACGAGACGTAGTCTTGCTGCTCTT
AAAAATATGGCCCATCATAAGCTACAAACCCTTGCAACTAACCTCTTGGCTTCTGAGGCA
TCTGACAAGGGAAATTTACCTAAATATTCTCATAACTCCCTGATGGTTCAAGCAATAAAG
ACAAACCTAACAGACCCGGACATACATGTGCTATTCTTTGATGTGGAAGCGTTGATTATT
CAACTCCTGACTGAAGAAGCCTCTAGGCCGAATACTGCTCTTATTTCCCCAGAGAATTTG
CAAAAAGCATCTGGCAGTTCAGACAAAGGGGGCTCTTTTTTAACTGGAAAACGAGCAGCA
GTTCTCTTCCAACAAGTGAAAGAAACGATCAAAGAGAACATCAAGGAACACCTCCTTGAT
GATGAAGAGGAGGATGAGGAGATAATGAGGCAGAGAAGGGAAGAAAGTGATCCTGAATAT
CGGTCCAGCAAATCAAAGCCATTGACCCTATTAGAATATAATTTAACTATGGACACTGCA
AAGCTGTTTATGTCCTGCCTTCACGCCTGGGGTTTGAATGAAGTACTGGATGAAGTTTGC
CTGGATCGCCTTGGAATGCTGAAACCCCACTGCACCGTATCGTTTGGCCTCTTGTCAAGA

GGAGGCCATATGTCACTGATGCTGCCGGGTTATAATCAGCCTGCTTGTAAACTGTCACAT
GGGAAAACAGAAGTAGGAAGGAAGCTGCCAGCGTCTGAGGGAGTAGGAAAGGGAACTTAC
GGAGTGTCCCGTGCCGTCACCACACAGCATCTCCTGTCTATCATTTCTTTGGCAAATACT
TTAATGAGTATGACCAATGCAACTTTTATTGGTGATCATATGAAGAAGGGTCCTACCAGG
CCACCTAGACCAAGCACCCCAGACCTTTCTAAGGCAAGGGGTTCCCCTCCAACTTCCAGT
AATATTGTGCAAGGACAGATTAAACAAGTTGCTGCACCTGTCGTTTCCGCTCGGTCTGAT
GCTGATCACTCTGGCTCTGACCCTCCTTCTGCTCCTGCTTTACATACCTGTTTCTTAGTA
AATGAAGGTTGGAGTCAGTTAGCTGCTATGCACTGTGTTATGCTGCCAGACCTACTGGGA
TTGGATAAATTTAGGCCTCCCCTTCTGGAGATGCTGGCCCGAAGATGGCAAGATCGATGC
TTGGAGGTGAGAGAAGCCGCGCAGGCCCTGCTTCTGGCGGAACTGAGAAGAATTGAGCAG
GCAGGCAGGAAGGAAGCCATTGATGCCTGGGCTCCTTACTTACCTCAGTACATAGACCAC
GTCATATCACCTGGAGTCACATCAGAAGCCGCGCAGACTATCACCACGGCTCCTGATGCC
TCAGGGCCTGAAGCAAAAGTCCAGGAGGAAGAGCATGACCTTGTTGACGATGACATCACC
ACTGGTTGCTTATCAAGTGTCCCACAAATGAAAAAAATTTCTACATCTTACGAGGAAAGA
CGGAAGCAAGCTACCGCTATTGTTTTACTTGGAGTAATAGGAGCTGAATTTGGTGCTGAA
ATTGAACCTCCTAAACTATTGACCAGACCTCGAAGCTCTAGCCAAATTCCTGAGGGATTC
GGGTTGACTAGTGGTGGATCCAACTACTCGCTGGCCAGACATACTTGCAAGGCACTGACG
TTTCTTCTGCTACAGCCTCCAAGCCCCAAACTTCCTCCACACAGCACTATCCGAAGAACA
GCCATTGATCTGATTGGACGTGGGTTCACTGTTTGGGAGCCTTACATGGATGTGTCCGCT
GTTCTGATGGGGCTTCTCGAACTTTGTGCCGATGCCGAGAAACAACTTGCCAACATCACA
ATGGGGTTGCCTCTGAGCCCAGCAGCTGACTCGGCCCGCTCTGCGAGGCATGCCCTCTCG
CTCATTGCCACCGCCAGACCACCCGCCTTCATCACCACCATAGCCAAAGAGGTACACAGA
CATACGGCTCTTGCAGCAAATACCCAATCACAGCAGAATATGCACACAACAACTCTTGCA
CGAGCTAAAGGGGAAATTTTGAGAGTCATTGAAATTCTTATTGAAAAGATGCCCACAGAT
GTTGTGGATCTTCTCGTGGAGGTTATGGACATCATTATGTACTGCCTTGAAGGATCTTTA
GTTAAAAAGAAAGGTCTTCAAGAATGTTTCCCAGCCATCTGCAGGTTCTACATGGTCAGC
TATTATGAGCGGAATCACAGAATAGCAGTTGGAGCTCGCCATGGTTCAGTGGCCCTGTAC
GACATCCGGACTGGAAAATGTCAGACAATCCATGGACACAAGGGACCAATCACTGCAGTG
GCTTTTGCTCCTGATGGAAGATATCTTGCCACCTACTCAAACACTGACAGCCACATTTCT
TTTTGGCAGATGAACACGTCACTGCTGGGAAGCATCGGCATGCTGAACTCGGCACCTCAG
CTGCGCTGCATTAAAACCTACCAGGTGCCCCCTGTGCAGCCCGCGTCCCCCGGCTCCCAC
AATGCCCTCAAGCTGGCCCGGCTCATCTGGACTTCCAACCGCAACGTCATCCTCATGGCC
CATGACGGGAAGGAGCACCGCTTCATGGTCTAA

B) Full amino acid sequence of the WDR7 protein in wild-type A549 cells: 1490 amino acid
MAGNSLVLPIVLWGRKAPTHCISAVLLTDDGATIVTGCHDGQICLWDLSVELQINPRALL
FGHTASITCLSKACASSDKQYIVSASESGEMCLWDVSDGRCIEFTKLACTHTGIQFYQFS
VGNQREGRLLCHGHYPEILVVDATSLEVLYSLVSKISPDWISSMSIIRSHRTQEDTVVAL
SVTGILKVWIVTSEISDMQDTEPIFEEESKPIYCQNCQSISFCAFTQRSLLVVCSKYWRV
FDAGDYSLLCSGPSENGQTWTGGDFVSSDKVIIWTENGQSYIYKLPASCLPASDSFRSDV
GKAVENLIPPVQHILLDRKDKELLICPPVTRFFYGCREYFHKLLIQGDSSGRLNIWNISD
TADKQGSEEGLAMTTSISLQEAFDKLNPCPAGIIDQLSVIPNSNEPLKVTASVYIPAHGR
LVCGREDGSIVIVPATQTAIVQLLQGEHMLRRGWPPHRTLRGHRNKVTCLLYPHQVSARY
DQRYLISGGVDFSVIIWDIFSGEMKHIFCVHGGEITQLLVPPENCSARVQHCICSVASDH
SVGLLSLREKKCIMLASRHLFPIQVIKWRPSDDYLVVGCSDGSVYVWQMDTGALDRCVMG
ITAVEILNACDEAVPAAVDSLSHPAVNLKQAMTRRSLAALKNMAHHKLQTLATNLLASEA
SDKGNLPKYSHNSLMVQAIKTNLTDPDIHVLFFDVEALIIQLLTEEASRPNTALISPENL
QKASGSSDKGGSFLTGKRAAVLFQQVKETIKENIKEHLLDDEEEDEEIMRQRREESDPEY
RSSKSKPLTLLEYNLTMDTAKLFMSCLHAWGLNEVLDEVCLDRLGMLKPHCTVSFGLLSR
GGHMSLMLPGYNQPACKLSHGKTEVGRKLPASEGVGKGTYGVSRAVTTQHLLSIISLANT
LMSMTNATFIGDHMKKGPTRPPRPSTPDLSKARGSPPTSSNIVQGQIKQVAAPVVSARSD
ADHSGSDPPSAPALHTCFLVNEGWSQLAAMHCVMLPDLLGLDKFRPPLLEMLARRWQDRC
LEVREAAQALLLAELRRIEQAGRKEAIDAWAPYLPQYIDHVISPGVTSEAAQTITTAPDA
SGPEAKVQEEEHDLVDDDITTGCLSSVPQMKKISTSYEERRKQATAIVLLGVIGAEFGAE
IEPPKLLTRPRSSSQIPEGFGLTSGGSNYSLARHTCKALTFLLLQPPSPKLPPHSTIRRT
AIDLIGRGFTVWEPYMDVSAVLMGLLELCADAEKQLANITMGLPLSPAADSARSARHALS

LIATARPPAFITTIAKEVHRHTALAANTQSQQNMHTTTLARAKGEILRVIEILIEKMPTD
VVDLLVEVMDIIMYCLEGSLVKKKGLQECFPAICRFYMVSYYERNHRIAVGARHGSVALY
DIRTGKCQTIHGHKGPITAVAFAPDGRYLATYSNTDSHISFWQMNTSLLGSIGMLNSAPQ
LRCIKTYQVPPVQPASPGSHNALKLARLIWTSNRNVILMAHDGKEHRFMV*

C) WDR7 KO cell line 1 (sgRNA 1): Indel 'A' insertion
ATGGCAGGAAACAGCCTTGTTCTACCCATTGTTCTTTGGGGTCGAAAAGCGCCCACACAT
TGCATCTCAGCCGTACTTTTAACAGATGATGGGGCCACGAAATCGTAACAGGATGTCACGAC
GGACAAATATGTCTCTGGGATCTTTCAGTAGAACTGCAAATTAATCCTCGAGCACTGTTG
TTTGGTCATACAGCATCAATCACTTGTTTGTCTAAAGCTTGTGCTTCCAGTGACAAACAG
TATATTGTGAGTGCATCTGAAAGTGGAGAGATGTGCCTCTGGGATGTGAGTGATGGCAGA
TGTATTGAATTTACAAAATTAGCTTGCACACATACTGGCATACAGTTCTACCAGTTCTCT
GTTGGGAATCAGCGAGAAGGAAGGCTTTTATGCCACGGACATTACCCTGAAATCCTTGTT
GTGGATGCTACCAGCCTTGAAGTATTATACTCCTTAGTATCAAAGATATCACCAGACTGG
ATTAGCTCCATGAGTATTATTCGATCCCACCGAACACAAGAGGACACAGTGGTAGCACTC
TCGGTGACTGGCATCCTGAAGGTCTGGATTGTTACCTCGGAAATAAGTGACATGCAGGAT
ACTGAGCCAATATTTGAGGAGGAATCCAAACCAATTTATTGTCAGAATTGCCAAAGCATC
TCTTTTTGTGCATTTACACAAAGGTCACTTTTGGTTGTGTGTTCCAAATATTGGAGGGTG
TTCGATGCCGGAGACTATTCCTTGTTGTGTTCAGGTCCTAGTGAAAATGGACAGACATGG
ACCGGGGGGGACTTTGTCTCATCAGATAAAGTCATCATTTGGACAGAAAATGGGCAAAGT
TATATTTACAAACTACCTGCCAGTTGCCTTCCAGCTAGTGATTCATTCCGCAGTGATGTG
GGGAAGGCAGTTGAAAATTTAATTCCTCCTGTACAACATATCCTCTTGGATCGAAAAGAT
AAAGAGTTGCTAATTTGTCCTCCTGTTACTCGGTTCTTCTATGGATGCAGAGAATATTTC
CATAAACTGTTAATTCAGGGTGATTCTTCTGGAAGGTTGAATATTTGGAACATATCAGAC
ACAGCTGATAAACAGGGAAGTGAAGAAGGGCTGGCAATGACAACTTCTATTAGTTTGCAA
GAGGCATTTGATAAACTGAATCCTTGTCCTGCTGGAATTATAGATCAGCTGAGTGTGATT
CCCAATAGTAATGAACCTCTTAAAGTAACTGCAAGTGTGTACATACCAGCACATGGACGA
CTTGTTTGTGGTCGTGAAGATGGAAGCATAGTTATTGTACCTGCCACACAGACGGCCATA
GTACAGCTGTTGCAAGGGGAACACATGCTCAGAAGAGGTTGGCCACCTCACAGAACACTC
CGTGGTCATCGGAACAAAGTCACATGTTTGCTATATCCTCATCAGGTCTCAGCTCGGTAT
GATCAAAGATACCTGATATCTGGAGGTGTGGATTTTTCAGTCATAATTTGGGACATATTT
TCTGGAGAAATGAAACATATCTTCTGTGTTCATGGTGGTGAGATTACTCAACTTCTAGTT
CCACCTGAAAACTGTAGTGCAAGAGTACAGCACTGCATCTGCTCTGTAGCCAGTGACCAC
TCAGTAGGACTTCTAAGTTTGCGAGAGAAAAAATGCATAATGTTGGCATCTCGTCACCTT
TTTCCTATTCAAGTAATCAAATGGAGGCCTTCTGATGATTACCTGGTGGTGGGGTGTTCA
GATGGTTCTGTGTACGTCTGGCAAATGGATACTGGTGCATTGGATCGTTGTGTGATGGGG
ATAACAGCAGTTGAGATTCTAAACGCTTGTGATGAAGCTGTTCCTGCTGCTGTTGATTCA
CTTAGTCATCCAGCAGTCAACCTAAAACAAGCTATGACGAGACGTAGTCTTGCTGCTCTT
AAAAATATGGCCCATCATAAGCTACAAACCCTTGCAACTAACCTCTTGGCTTCTGAGGCA
TCTGACAAGGGAAATTTACCTAAATATTCTCATAACTCCCTGATGGTTCAAGCAATAAAG
ACAAACCTAACAGACCCGGACATACATGTGCTATTCTTTGATGTGGAAGCGTTGATTATT
CAACTCCTGACTGAAGAAGCCTCTAGGCCGAATACTGCTCTTATTTCCCCAGAGAATTTG
CAAAAAGCATCTGGCAGTTCAGACAAAGGGGGCTCTTTTTAACTGGAAAACGAGCAGCA
GTTCTCTTCCAACAAGTGAAAGAAACGATCAAAGAGAACATCAAGGAACACCTCCTTGAT
GATGAAGAGGAGGATGAGGAGATAATGAGGCAGAGAAGGGAAGAAAGTGATCCTGAATAT
CGGTCCAGCAAATCAAAGCCATTGACCCTATTAGAATATAATTTAACTATGGACACTGCA
AAGCTGTTTATGTCCTGCCTTCACGCCTGGGGTTTGAATGAAGTACTGGATGAAGTTTGC
CTGGATCGCCTTGGAATGCTGAAACCCCACTGCACCGTATCGTTTGGCCTCTTGTCAAGA
GGAGGCCATATGTCACTGATGCTGCCGGGTTATAATCAGCCTGCTTGTAAACTGTCACAT
GGGAAAACAGAAGTAGGAAGGAAGCTGCCAGCGTCTGAGGGAGTAGGAAAGGGAACTTAC
GGAGTGTCCCGTGCCGTCACCACACAGCATCTCCTGTCTATCATTTCTTTGGCAAATACT
TTAATGAGTATGACCAATGCAACTTTTATTGGTGATCATATGAAGAAGGGTCCTACCAGG
CCACCTAGACCAAGCACCCCAGACCTTTCTAAGGCAAGGGGTTCCCCTCCAACTTCCAGT
AATATTGTGCAAGGACAGATTAAACAAGTTGCTGCACCTGTCGTTTCCGCTCGGTCTGAT
GCTGATCACTCTGGCTCTGACCCTCCTTCTGCTCCTGCTTTACATACCTGTTTCTTAGTA

AATGAAGGTTGGAGTCAGTTAGCTGCTATGCACTGTGTTATGCTGCCAGACCTACTGGGA
TTGGATAAATTTAGGCCTCCCCTTCTGGAGATGCTGGCCCGAAGATGGCAAGATCGATGC
TTGGAGGTGAGAGAAGCCGCGCAGGCCCTGCTTCTGGCGGAACTGAGAAGAATTGAGCAG
GCAGGCAGGAAGGAAGCCATTGATGCCTGGGCTCCTTACTTACCTCAGTACATAGACCAC
GTCATATCACCTGGAGTCACATCAGAAGCCGCGCAGACTATCACCACGGCTCCTGATGCC
TCAGGGCCTGAAGCAAAAGTCCAGGAGGAAGAGCATGACCTTGTTGACGATGACATCACC
ACTGGTTGCTTATCAAGTGTCCCACAAATGAAAAAAATTTCTACATCTTACGAGGAAAGA
CGGAAGCAAGCTACCGCTATTGTTTTACTTGGAGTAATAGGAGCTGAATTTGGTGCTGAA
ATTGAACCTCCTAAACTATTGACCAGACCTCGAAGCTCTAGCCAAATTCCTGAGGGATTC
GGGTTGACTAGTGGTGGATCCAACTACTCGCTGGCCAGACATACTTGCAAGGCACTGACG
TTTCTTCTGCTACAGCCTCCAAGCCCCAAACTTCCTCCACACAGCACTATCCGAAGAACA
GCCATTGATCTGATTGGACGTGGGTTCACTGTTTGGGAGCCTTACATGGATGTGTCCGCT
GTTCTGATGGGGCTTCTCGAACTTTGTGCCGATGCCGAGAAACAACTTGCCAACATCACA
ATGGGGTTGCCTCTGAGCCCAGCAGCTGACTCGGCCCGCTCTGCGAGGCATGCCCTCTCG
CTCATTGCCACCGCCAGACCACCCGCCTTCATCACCACCATAGCCAAAGAGGTACACAGA
CATACGGCTCTTGCAGCAAATACCCAATCACAGCAGAATATGCACACAACAACTCTTGCA
CGAGCTAAAGGGGAAATTTTGAGAGTCATTGAAATTCTTATTGAAAAGATGCCCACAGAT
GTTGTGGATCTTCTCGTGGAGGTTATGGACATCATTATGTACTGCCTTGAAGGATCTTTA
GTTAAAAAGAAAGGTCTTCAAGAATGTTTCCCAGCCATCTGCAGGTTCTACATGGTCAGC
TATTATGAGCGGAATCACAGAATAGCAGTTGGAGCTCGCCATGGTTCAGTGGCCCTGTAC
GACATCCGGACTGGAAAATGTCAGACAATCCATGGACACAAGGGACCAATCACTGCAGTG
GCTTTTGCTCCTGATGGAAGATATCTTGCCACCTACTCAAACACTGACAGCCACATTTCT
TTTTGGCAGATGAACACGTCACTGCTGGGAAGCATCGGCATGCTGAACTCGGCACCTCAG
CTGCGCTGCATTAAAACCTACCAGGTGCCCCCTGTGCAGCCCGCGTCCCCCGGCTCCCAC
AATGCCCTCAAGCTGGCCCGGCTCATCTGGACTTCCAACCGCAACGTCATCCTCATGGCC
CATGACGGGAAGGAGCACCGCTTCATGGTCTAA

D) Full amino acid sequence: WDR7 protein-frame shift mutation (stop codon) early termination:
MAGNSLVLPIVLWGRKAPTHCISAVLLTDDGATNRNRMSRRTNMSLGSFSRTAN*

E) WDR7 KO cell line 1 (sgRNA1): Indel ‘T’ deletion
ATGGCAGGAAACAGCCTTGTTCTACCCATTGTTCTTTGGGGTCGAAAAGCGCCCACACAT
TGCATCTCAGCCGTACTTTTAACAGATGATGGGGCCACGA_CGTAACAGGATGTCACGAC
GGACAAATATGTCTCTGGGATCTTTCAGTAGAACTGCAAATTAATCCTCGAGCACTGTTG
TTTGGTCATACAGCATCAATCACTTGTTTGTCTAAAGCTTGTGCTTCCAGTGACAAACAG
TATATTGTGAGTGCATCTGAAAGTGGAGAGATGTGCCTCTGGGATGTGAGTGATGGCAGA
TGTATTGAATTTACAAAATTAGCTTGCACACATACTGGCATACAGTTCTACCAGTTCTCT
GTTGGGAATCAGCGAGAAGGAAGGCTTTTATGCCACGGACATTACCCTGAAATCCTTGTT
GTGGATGCTACCAGCCTTGAAGTATTATACTCCTTAGTATCAAAGATATCACCAGACTGG
ATTAGCTCCATGAGTATTATTCGATCCCACCGAACACAAGAGGACACAGTGGTAGCACTC
TCGGTGACTGGCATCCTGAAGGTCTGGATTGTTACCTCGGAAATAAGTGACATGCAGGAT
ACTGAGCCAATATTTGAGGAGGAATCCAAACCAATTTATTGTCAGAATTGCCAAAGCATC
TCTTTTTGTGCATTTACACAAAGGTCACTTTTGGTTGTGTGTTCCAAATATTGGAGGGTG
TTCGATGCCGGAGACTATTCCTTGTTGTGTTCAGGTCCTAGTGAAAATGGACAGACATGG
ACCGGGGGGGACTTTGTCTCATCAGATAAAGTCATCATTTGGACAGAAAATGGGCAAAGT
TATATTTACAAACTACCTGCCAGTTGCCTTCCAGCTAGTGATTCATTCCGCAGTGATGTG
GGGAAGGCAGTTGAAAATTTAATTCCTCCTGTACAACATATCCTCTTGGATCGAAAAGAT
AAAGAGTTGCTAATTTGTCCTCCTGTTACTCGGTTCTTCTATGGATGCAGAGAATATTTC
CATAAACTGTTAATTCAGGGTGATTCTTCTGGAAGGTTGAATATTTGGAACATATCAGAC
ACAGCTGATAAACAGGGAAGTGAAGAAGGGCTGGCAATGACAACTTCTATTAGTTTGCAA
GAGGCATTTGATAAACTGAATCCTTGTCCTGCTGGAATTATAGATCAGCTGAGTGTGATT
CCCAATAGTAATGAACCTCTTAAAGTAACTGCAAGTGTGTACATACCAGCACATGGACGA
CTTGTTTGTGGTCGTGAAGATGGAAGCATAGTTATTGTACCTGCCACACAGACGGCCATA
GTACAGCTGTTGCAAGGGGAACACATGCTCAGAAGAGGTTGGCCACCTCACAGAACACTC

CGTGGTCATCGGAACAAAGTCACATGTTTGCTATATCCTCATCAGGTCTCAGCTCGGTAT
GATCAAAGATACCTGATATCTGGAGGTGTGGATTTTTCAGTCATAATTTGGGACATATTT
TCTGGAGAAATGAAACATATCTTCTGTGTTCATGGTGGTGAGATTACTCAACTTCTAGTT
CCACCTGAAAACTGTAGTGCAAGAGTACAGCACTGCATCTGCTCTGTAGCCAGTGACCAC
TCAGTAGGACTTCTAAGTTTGCGAGAGAAAAAATGCATAATGTTGGCATCTCGTCACCTT
TTTCCTATTCAAGTAATCAAATGGAGGCCTTCTGATGATTACCTGGTGGTGGGGTGTTCA
GATGGTTCTGTGTACGTCTGGCAAATGGATACTGGTGCATTGGATCGTTGTGTGATGGGG
ATAACAGCAGTTGAGATTCTAAACGCTTGTGATGAAGCTGTTCCTGCTGCTGTTGATTCA
CTTAGTCATCCAGCAGTCAACCTAAAACAAGCTATGACGAGACGTAGTCTTGCTGCTCTT
AAAAATATGGCCCATCATAAGCTACAAACCCTTGCAACTAACCTCTTGGCTTCTGAGGCA
TCTGACAAGGGAAATTTACCTAAATATTCTCATAACTCCCTGATGGTTCAAGCAATAAAG
ACAAACCTAACAGACCCGGACATACATGTGCTATTCTTTGATGTGGAAGCGTTGATTATT
CAACTCCTGACTGAAGAAGCCTCTAGGCCGAATACTGCTCTTATTTCCCCAGAGAATTTG
CAAAAAGCATCTGGCAGTTCAGACAAAGGGGGCTCTTTTTAACTGGAAAACGAGCAGCA
GTTCTCTTCCAACAAGTGAAAGAAACGATCAAAGAGAACATCAAGGAACACCTCCTTGAT
GATGAAGAGGAGGATGAGGAGATAATGAGGCAGAGAAGGGAAGAAAGTGATCCTGAATAT
CGGTCCAGCAAATCAAAGCCATTGACCCTATTAGAATATAATTTAACTATGGACACTGCA
AAGCTGTTTATGTCCTGCCTTCACGCCTGGGGTTTGAATGAAGTACTGGATGAAGTTTGC
CTGGATCGCCTTGGAATGCTGAAACCCCACTGCACCGTATCGTTTGGCCTCTTGTCAAGA
GGAGGCCATATGTCACTGATGCTGCCGGGTTATAATCAGCCTGCTTGTAAACTGTCACAT
GGGAAAACAGAAGTAGGAAGGAAGCTGCCAGCGTCTGAGGGAGTAGGAAAGGGAACTTAC
GGAGTGTCCCGTGCCGTCACCACACAGCATCTCCTGTCTATCATTTCTTTGGCAAATACT
TTAATGAGTATGACCAATGCAACTTTTATTGGTGATCATATGAAGAAGGGTCCTACCAGG
CCACCTAGACCAAGCACCCCAGACCTTTCTAAGGCAAGGGGTTCCCCTCCAACTTCCAGT
AATATTGTGCAAGGACAGATTAAACAAGTTGCTGCACCTGTCGTTTCCGCTCGGTCTGAT
GCTGATCACTCTGGCTCTGACCCTCCTTCTGCTCCTGCTTTACATACCTGTTTCTTAGTA
AATGAAGGTTGGAGTCAGTTAGCTGCTATGCACTGTGTTATGCTGCCAGACCTACTGGGA
TTGGATAAATTTAGGCCTCCCCTTCTGGAGATGCTGGCCCGAAGATGGCAAGATCGATGC
TTGGAGGTGAGAGAAGCCGCGCAGGCCCTGCTTCTGGCGGAACTGAGAAGAATTGAGCAG
GCAGGCAGGAAGGAAGCCATTGATGCCTGGGCTCCTTACTTACCTCAGTACATAGACCAC
GTCATATCACCTGGAGTCACATCAGAAGCCGCGCAGACTATCACCACGGCTCCTGATGCC
TCAGGGCCTGAAGCAAAAGTCCAGGAGGAAGAGCATGACCTTGTTGACGATGACATCACC
ACTGGTTGCTTATCAAGTGTCCCACAAATGAAAAAAATTTCTACATCTTACGAGGAAAGA
CGGAAGCAAGCTACCGCTATTGTTTTACTTGGAGTAATAGGAGCTGAATTTGGTGCTGAA
ATTGAACCTCCTAAACTATTGACCAGACCTCGAAGCTCTAGCCAAATTCCTGAGGGATTC
GGGTTGACTAGTGGTGGATCCAACTACTCGCTGGCCAGACATACTTGCAAGGCACTGACG
TTTCTTCTGCTACAGCCTCCAAGCCCCAAACTTCCTCCACACAGCACTATCCGAAGAACA
GCCATTGATCTGATTGGACGTGGGTTCACTGTTTGGGAGCCTTACATGGATGTGTCCGCT
GTTCTGATGGGGCTTCTCGAACTTTGTGCCGATGCCGAGAAACAACTTGCCAACATCACA
ATGGGGTTGCCTCTGAGCCCAGCAGCTGACTCGGCCCGCTCTGCGAGGCATGCCCTCTCG
CTCATTGCCACCGCCAGACCACCCGCCTTCATCACCACCATAGCCAAAGAGGTACACAGA
CATACGGCTCTTGCAGCAAATACCCAATCACAGCAGAATATGCACACAACAACTCTTGCA
CGAGCTAAAGGGGAAATTTTGAGAGTCATTGAAATTCTTATTGAAAAGATGCCCACAGAT
GTTGTGGATCTTCTCGTGGAGGTTATGGACATCATTATGTACTGCCTTGAAGGATCTTTA
GTTAAAAAGAAAGGTCTTCAAGAATGTTTCCCAGCCATCTGCAGGTTCTACATGGTCAGC
TATTATGAGCGGAATCACAGAATAGCAGTTGGAGCTCGCCATGGTTCAGTGGCCCTGTAC
GACATCCGGACTGGAAAATGTCAGACAATCCATGGACACAAGGGACCAATCACTGCAGTG
GCTTTTGCTCCTGATGGAAGATATCTTGCCACCTACTCAAACACTGACAGCCACATTTCT
TTTTGGCAGATGAACACGTCACTGCTGGGAAGCATCGGCATGCTGAACTCGGCACCTCAG
CTGCGCTGCATTAAAACCTACCAGGTGCCCCCTGTGCAGCCCGCGTCCCCCGGCTCCCAC
AATGCCCTCAAGCTGGCCCGGCTCATCTGGACTTCCAACCGCAACGTCATCCTCATGGCC
CATGACGGGAAGGAGCACCGCTTCATGGTCTAA

F) Amino acid sequence: WDR7 protein (stop codon) early termination

MAGNSLVLPIVLWGRKAPTHCISAVLLTDDGATT*

G) WDR7 KO cell line 2 (sgRNA 5): Indel 'T' insertion

ATGGCAGGAAACAGCCTTGTTCTACCCATTGTTCTTTGGGGTCGAAAAGCGCCCACACATTGCATCTCA
GCCGTACTTTTAACAGATGATGGGGCCACGATCGTAACAGGATGTCACGACGGACAAATATGTCTCTG
GGATCTTTCAGTAGAACTGCAAATTAATCCTCGAGCACTGTTGTTTGGTCATACAGCATCAATCACTTG
TTTGTCTAAAGCTTGTGCTTCCAGTGACAAACAGTATATTGTGAGTGCATCTGAAAGTGGAGAGATGT
GCCTCTGGGATGTGAGTGATGGCAGATGTATTGAATTTACAAAATTAGCTTGCACACATACTGGCATA
CAGTTCTACCAGTTCTCTGTTGGGAATCAGCGAGAAGGAAGGCTTTTATGCCACGGACATTACCCTGA
AATCCTTGTTGTGGATGCTACCAGCCTTGAAGTATTATACTCCTTAGTATCAAAGATATCACCAGACTG
GATTAGCTCCATGAGTATTATTCGATCCCACCGAACACAAGAGGACACAGTGGTAGCACTCTCGGTGA
CTGGCATCCTGAAGGTCTGGATTGTTACCTCGGAAATAAGTGACATGCAGGATACTGAGCCAATATTT
GAGGAGGAATCCAAACCAATTTATTGTCAGAATTGCCAAAGCATCTCTTTTTGTGCATTTACACAAAG
GTCACTTTTGGTTGTGTGTTCCAAATATTGGAGGGTGTTCGATGCCGGAGACTATTCCTTGTTGTGTTC
AGGTCCTAGTGAAAATGGACAGACATGGACCGGGGGGGACTTTGTCTCATCAGATAAAGTCATCATTT
GGACAGAAAATGGGCAAAGTTATATTTACAAACTACCTGCCAGTTGCCTTCCAGCTAGTGATTCATTC
CGCAGTGATGTGGGGAAGGCAGTTGAAAATTTAATTCCTCCTGTACAACATATCCTCTTGGATCGAAA
AGATAAAGAGTTGCTAATTTGTCCTCCTGTTACTCGGTTCTTCTATGGATGCAGAGAATATTTCCATAA
ACTGTTAATTCAGGGTGATTCTTCTGGAAGGTTGAATATTTGGAACATATCAGACACAGCTGATAAAC
AGGGAAGTGAAGAAGGGCTGGCAATGACAACTTCTATTAGTTTGCAAGAGGCATTTGATAAACTGAAT
CCTTGTCCTGCTGGAATTATAGATCAGCTGAGTGTGATTCCCAATAGTAATGAACCTCTTAAAGTAACT
GCAAGTGTGTACATACCAGCACATGGACGACTTGTTTGTGGTCGTGAAGATGGAAGCATAGTTATTGT
ACCTGCCACACAGACGGCCATAGTACAGCTGTTGCAAGGGGAACACATGCTCAGAAGAGGTTGGCCA
CCTCACAGAACACTCCGTGGTCATCGGAACAAAGTCACATGTTTGCTATATCCTCATCAGGTCTCAGCT
CGGTATGATCAAAGATACCTGATATCTGGAGGTGTGGATTTTTCAGTCATAATTTGGGACATATTTTCT
GGAGAAATGAAACATATCTTCTGTGTTCATGGTGGTGAGATTACTCAACTTCTAGTTCCACCTGAAAAC
TGTAGTGCAAGAGTACAGCACTGCATCTGCTCTGTAGCCAGTGACCACTCAGTAGGACTTCTAAGTTT
GCGAGAGAAAAAAATGCATAATGTTGGCATCTCGTCACCTTTTTCCTATTCAAGTAATCAAATGGAGGC
CTTCTGATGATTACCTGGTGGTGGGGTGTTCAGATGGTTCTGTGTACGTCTGGCAAATGGATACTGGTG
CATTGGATCGTTGTGTGATGGGGATAACAGCAGTTGAGATTCTAAACGCTTGTGATGAAGCTGTTCCT
GCTGCTGTTGATTCACTTAGTCATCCAGCAGTCAACCTAAAACAAGCTATGACGAGACGTAGTCTTGCT
GCTCTTAAAAATATGGCCCATCATAAGCTACAAACCCTTGCAACTAACCTCTTGGCTTCTGAGGCATCT
GACAAGGGAAATTTACCTAAATATTCTCATAACTCCCTGATGGTTCAAGCAATAAAGACAAACCTAAC
AGACCCGGACATACATGTGCTATTCTTTGATGTGGAAGCGTTGATTATTCAACTCCTGACTGAAGAAG
CCTCTAGGCCGAATACTGCTCTTATTTCCCCAGAGAATTTGCAAAAAGCATCTGGCAGTTCAGACAAA
GGGGGCTCTTTTTTAACTGGAAAACGAGCAGCAGTTCTCTTCCAACAAGTGAAAGAAACGATCAAAGA
GAACATCAAGGAACACCTCCTTGATGATGAAGAGGAGGATGAGGAGATAATGAGGCAGAGAAGGGA
AGAAAGTGATCCTGAATATCGGTCCAGCAAATCAAAGCCATTGACCCTATTAGAATATAATTTAACTA
TGGACACTGCAAAGCTGTTTATGTCCTGCCTTCACGCCTGGGGTTTGAATGAAGTACTGGATGAAGTTT
GCCTGGATCGCCTTGGAATGCTGAAACCCCACTGCACCGTATCGTTTGGCCTCTTGTCAAGAGGAGGC
CATATGTCACTGATGCTGCCGGGTTATAATCAGCCTGCTTGTAAACTGTCACATGGGAAAACAGAAGT
AGGAAGGAAGCTGCCAGCGTCTGAGGGAGTAGGAAAGGGAACTTACGGAGTGTCCCGTGCCGTCACC
ACACAGCATCTCCTGTCTATCATTTCTTTGGCAAATACTTTAATGAGTATGACCAATGCAACTTTTATT
GGTGATCATATGAAGAAGGGTCCTACCAGGCCACCTAGACCAAGCACCCCAGACCTTTCTAAGGCAAG
GGGTTCCCCTCCAACTTCCAGTAATATTGTGCAAGGACAGATTAAACAAGTTGCTGCACCTGTCGTTTC
CGCTCGGTCTGATGCTGATCACTCTGGCTCTGACCCTCCTTCTGCTCCTGCTTTACATACCTGTTTCTTA
GTAAATGAAGGTTGGAGTCAGTTAGCTGCTATGCACTGTGTTATGCTGCCAGACCTACTGGGATTGGA
TAAATTTAGGCCTCCCCTTCTGGAGATGCTGGCCCGAAGATTGGCAAGATCGATGCTTGGAGGTGAGA
GAAGCCGCGCAGGCCCTGCTTCTGGCGGAACTGAGAAGAATTGAGCAGGCAGGCAGGAAGGAAGCCA
TTGATGCCTGGGCTCCTTACTTACCTCAGTACATAGACCACGTCATATCACCTGGAGTCACATCAGAAG
CCGCGCAGACTATCACCACGGCTCCTGATGCCTCAGGGCCTGAAGCAAAAGTCCAGGAGGAAGAGCA
TGACCTTGTTGACGATGACATCACCACTGGTTGCTTATCAAGTGTCCCACAAATGAAAAAAATTTCTAC
ATCTTACGAGGAAAGACGGAAGCAAGCTACCGCTATTGTTTTACTTGGAGTAATAGGAGCTGAATTTG
GTGCTGAAATTGAACCTCCTAAACTATTGACCAGACCTCGAAGCTCTAGCCAAATTCCTGAGGGATTC
GGGTTGACTAGTGGTGGATCCAACTACTCGCTGGCCAGACATACTTGCAAGGCACTGACGTTTCTTCTG
CTACAGCCTCCAAGCCCCAAACTTCCTCCACACAGCACTATCCGAAGAACAGCCATTGATCTGATTGG
ACGTGGGTTCACTGTTTGGGAGCCTTACATGGATGTGTCCGCTGTTCTGATGGGGCTTCTCGAACTTTG

TGCCGATGCCGAGAAACAACTTGCCAACATCACAATGGGGTTGCCTCTGAGCCCAGCAGCTGACTCGG
CCCGCTCTGCGAGGCATGCCCTCTCGCTCATTGCCACCGCCAGACCACCCGCCTTCATCACCACCATAG
CCAAAGAGGTACACAGACATACGGCTCTTGCAGCAAATACCCAATCACAGCAGAATATGCACACAAC
AACTCTTGCACGAGCTAAAGGGGAAATTTTGAGAGTCATTGAAATTCTTATTGAAAAGATGCCCACAG
ATGTTGTGGATCTTCTCGTGGAGGTTATGGACATCATTATGTACTGCCTTGAAGGATCTTTAGTTAAAA
AGAAAGGTCTTCAAGAATGTTTCCCAGCCATCTGCAGGTTCTACATGGTCAGCTATTATGAGCGGAAT
CACAGAATAGCAGTTGGAGCTCGCCATGGTTCAGTGGCCCTGTACGACATCCGGACTGGAAAATGTCA
GACAATCCATGGACACAAGGGACCAATCACTGCAGTGGCTTTTGCTCCTGATGGAAGATATCTTGCCA
CCTACTCAAACACTGACAGCCACATTTCTTTTTGGCAGATGAACACGTCACTGCTGGGAAGCATCGGC
ATGCTGAACTCGGCACCTCAGCTGCGCTGCATTAAAACCTACCAGGTGCCCCCTGTGCAGCCCGCGTC
CCCCGGCTCCCACAATGCCCTCAAGCTGGCCCGGCTCATCTGGACTTCCAACCGCAACGTCATCCTCAT
GGCCCATGACGGGAAGGAGCACCGCTTATGGTCTAATGCTGCTGCCTGCCGCCGTGACTGCGTTTTAG
TTCTCTAAATTATCCAAGCCGATGTTGCTCTGTCCTTCCTCACACCAGATTGTTCCCAGGGGCCTGCCC
ACCCCAGTGCCATCCAGTGGCACGGCCGGGTCTTGTCACTTGTGCATGCTTCTCAGGGGCAGAACCCG
CTCGTGCCATCTGTCGATTCAGAGGCACGCACACATGCTCTGCAGGATGTGGCCATCCTGTCACCAGG
TAGTTTTTCCCTGCCAGAGATGGCAGGGGAAAGCCAGTGGTTCCTGGGAACGCTCTTGTTGCTTGGTGC
AACAGAAGCACAAAAATCAATAAACACTGTGATTGCACTCCCAGCCCAGATCAGCATTTTTAGCCATC
TCAACCGCACCTCTGAAGTGCTGCTTGAATCTGGCCGTTCTGACACTGGGTGTTGAGGTCATGTGGCAG
TCCTCACGATTGAAATTATTGCCCTTTGAATTACCTAAAATATTGTTTACCAAAAAGATTCTGTGTTTA
CATGTTCTTTTCTTCTGGTGATTGCTAGGAAATTACTCAATCCAATTTCCTGGGTTTAGCTACCACCTTC
TTTATCAACAGCTAACCAATTTGAGATTCATGATTAATTATGTACACAAAAATATTTCCTTGTAGAAA
ATAGTTTTCCAGACACTTAGCCTTCTGAAGTGCTCATCTCTTGCTTAAATGATAGTCTATTTAGTAGTTT
AAAATAAAAATAATCACTCCCTATGATCTAGTTAATGTAATTATCCAGTCACTGTAACTAATCTCTTGA
AGGGACTTTTGTGATAAAGAGCCAAGTTCAGAAGCATAGCGTGTACCGAAAATGAAGACTCTCCTATC
TACTGCTACAGATCCTGTTGAAGAAAGAGCTTACCCCTTTCCCCCGGAAAACTTGCAATCCTGTAGTTC
CCATATGTGTGGCATATGTCAGTTATGAAATTATTATAATTCAGCCTTCAAACTACTACTGATCAACTA
AAGGAAAGCTAATAAGGTATTTCTTTTAAACAAGTATTCTGGAAAAGTAATAACTATGGCACAAATGT
TCAGGAGTGTATATAAAAATATGTGGGCTCTCGATTTATTTGAGCAAACATTGCAATGAGGGTGTTCA
TTATGCTTCTAAGTAAGCTTTAACTAGACCACTTTCTTCTTATGTCAGATTGTGCTGGTTCTAGCTCATA
ATAATGAAAAAAATAGAATTATATTTTGTATGAATTGTTTCTAACAAACCTTGATCTGTATATACTTGT
GAGGGGTTGTTTGGTTGCCTAATTGTTGATTGGTTGGTTAGTTGGGCAAAATCATTTATTACATGCAAA
AATAGAAAATTATTTGTTAAATTCAGAGAAAATATGTGAAGTTTACAAAGGAACCAAAATGATAATGG
GAGAATTTAGTCTTAAAAGTTTAGGTTGTCAGAGTTTATCTGATATACAAAAAAGTAGGATCAAAGTC
TAGGAACAAACATTGGTTATTTCACTGAAACAGATTTTTAAAGAGCATATTAAGAAATTAAGACCATT
AGATCATCTCTTTCATAATGATATGATGTTATTTCCATTGACGGCAAATCTATACTGTTCCTTGGTTTTA
GAGTTGGGTATTCTTTCAAAAGTGAATTTAGGCACATGCATGTACATTGTATTTATAAAGATTATATAT
GTAGTTTCCAAGGAAGAAACAAAGACCTATACAGTTTAGACTTTATTTGTTTGGTGAACGTGCTGTTGT
GTTTGGCATAGCTAGAGAAATGTAAGGTAAAAGGCTCACTTTGTTTTCCAGTGGGTCCAGAGTCTTCTC
TATACCCAACATATGTTTACTTTATGAACTTAAATGGAAATTAATTTATTGTAGACTACATCTCTATTTG
GAATGATGAGGTAAATGTATACCTTAGCATTTTCTTTAACAAAAATGTTCTGTGTGTCAGGAAAGAGG
TGTGAGAGTGGTAGGCAGCCTGCCGCAAACACACGGATGGCTCCCCCCGCCACGAAGGTTGAGAACA
AAGCCAGGGGGCCCAGAGGAGCAATAGCCCTGGGCCACAAGCGTGGGTAGGCCTTTCACGTATTCCG
TCTGCACAGGCAACCAAGGCCAGTAGAAAGCTATGGCTGCAAAACCCTGGGGTGGACGATGTTTGAT
GATTAGACGGTCATCTCTAACTACTAAACTAATATCTGCATTCTTGTTAACATAAATCTCTCCTTGGCT
ATTTTAATAGCCACAGTCCCAAACTTCATCCCAGCTTAGATTTCTCAGCACTGAGGTAAACTCTGCATT
GTCATCCCAGCGTACACCTTTAATGTTTTATTGATTTCACTGGCACTGTATTTGGACCTGTCCTTGTATA
TGTAGAGACATATGTGGCTTCATTGGTAATTTACAAGCAAAAGATGACATGACGTGACACCTGTATGA
AAATGTGATGATTGAACTCCCTGTGTACAGCGAGTGAATTAAAATGTCTGCCTCTCCAGACATTTCTTA
ATGATTCCACTTAATAGACTCTATGTGTGCTGAATGTTCCTGTGTACATATGTGTGTTAAATAAAACAA
TTGTATTGAA

H) Amino acid sequence: WDR7 protein truncation (1039 aa)
MAGNSLVLPIVLWGRKAPTHCISAVLLTDDGATIVTGCHDGQICLWDLSVELQINPRALL
FGHTASITCLSKACASSDKQYIVSASESGEMCLWDVSDGRCIEFTKLACTHTGIQFYQFS
VGNQREGRLLCHGHYPEILVVDATSLEVLYSLVSKISPDWISSMSIIRSHRTQEDTVVAL
SVTGILKVWIVTSEISDMQDTEPIFEEESKPIYCQNCQSISFCAFTQRSLLVVCSKYWRV

FDAGDYSLLCSGPSENGQTWTGGDFVSSDKVIIWTENGQSYIYKLPASCLPASDSFRSDV
GKAVENLIPPVQHILLDRKDKELLICPPVTRFFYGCREYFHKLLIQGDSSGRLNIWNISD
TADKQGSEEGLAMTTSISLQEAFDKLNPCPAGIIDQLSVIPNSNEPLKVTASVYIPAHGR
LVCGREDGSIVIVPATQTAIVQLLQGEHMLRRGWPPHRTLRGHRNKVTCLLYPHQVSARY
DQRYLISGGVDFSVIIWDIFSGEMKHIFCVHGGEITQLLVPPENCSARVQHCICSVASDH
SVGLLSLREKKCIMLASRHLFPIQVIKWRPSDDYLVVGCSDGSVYVWQMDTGALDRCVMG
ITAVEILNACDEAVPAAVDSLSHPAVNLKQAMTRRSLAALKNMAHHKLQTLATNLLASEA
SDKGNLPKYSHNSLMVQAIKTNLTDPDIHVLFFDVEALIIQLLTEEASRPNTALISPENL
QKASGSSDKGGSFLTGKRAAVLFQQVKETIKENIKEHLLDDEEEDEEIMRQRREESDPEY
RSSKSKPLTLLEYNLTMDTAKLFMSCLHAWGLNEVLDEVCLDRLGMLKPHCTVSFGLLSR
GGHMSLMLPGYNQPACKLSHGKTEVGRKLPASEGVGKGTYGVSRAVTTQHLLSIISLANT
LMSMTNATFIGDHMKKGPTRPPRPSTPDLSKARGSPPTSSNIVQGQIKQVAAPVVSARSD
ADHSGSDPPSAPALHTCFLVNEGWSQLAAMHCVMLPDLLGLDKFRPPLLEMLARRLARSM
LGGERSRAGPASGGTEKN*

I) WDR7 KO cell line 2 (sgRNA 5): Indel ‘G’ deletion
ATGGCAGGAAACAGCCTTGTTCTACCCATTGTTCTTGGGGTCGAAAAGCGCCCACACATTGCATCTCA
GCCGTACTTTTAACAGATGATGGGGCCACGATCGTAACAGGATGTCACGACGGACAAATATGTCTCTG
GGATCTTTCAGTAGAACTGCAAATTAATCCTCGAGCACTGTTGTTTGGTCATACAGCATCAATCACTTG
TTTGTCTAAAGCTTGTGCTTCCAGTGACAAACAGTATATTGTGAGTGCATCTGAAAGTGGAGAGATGT
GCCTCTGGGATGTGAGTGATGGCAGATGTATTGAATTTACAAAATTAGCTTGCACACATACTGGCATA
CAGTTCTACCAGTTCTCTGTTGGGAATCAGCGAGAAGGAAGGCTTTTATGCCACGGACATTACCCTGA
AATCCTTGTTGTGGATGCTACCAGCCTTGAAGTATTATACTCCTTAGTATCAAAGATATCACCAGACTG
GATTAGCTCCATGAGTATTATTCGATCCCACCGAACACAAGAGGACACAGTGGTAGCACTCTCGGTGA
CTGGCATCCTGAAGGTCTGGATTGTTACCTCGGAAATAAGTGACATGCAGGATACTGAGCCAATATTT
GAGGAGGAATCCAAACCAATTTATTGTCAGAATTGCCAAAGCATCTCTTTTTGTGCATTTACACAAAG
GTCACTTTTGGTTGTGTGTTCCAAATATTGGAGGGTGTTCGATGCCGGAGACTATTCCTTGTTGTGTTC
AGGTCCTAGTGAAAATGGACAGACATGGACCGGGGGGGACTTTGTCTCATCAGATAAAGTCATCATTT
GGACAGAAAATGGGCAAAGTTATATTTACAAACTACCTGCCAGTTGCCTTCCAGCTAGTGATTCATTC
CGCAGTGATGTGGGGAAGGCAGTTGAAAATTTAATTCCTCCTGTACAACATATCCTCTTGGATCGAAA
AGATAAAGAGTTGCTAATTTGTCCTCCTGTTACTCGGTTCTTCTATGGATGCAGAGAATATTTCCATAA
ACTGTTAATTCAGGGTGATTCTTCTGGAAGGTTGAATATTTGGAACATATCAGACACAGCTGATAAAC
AGGGAAGTGAAGAAGGGCTGGCAATGACAACTTCTATTAGTTTGCAAGAGGCATTTGATAAACTGAAT
CCTTGTCCTGCTGGAATTATAGATCAGCTGAGTGTGATTCCCAATAGTAATGAACCTCTTAAAGTAACT
GCAAGTGTGTACATACCAGCACATGGACGACTTGTTTGTGGTCGTGAAGATGGAAGCATAGTTATTGT
ACCTGCCACACAGACGGCCATAGTACAGCTGTTGCAAGGGGAACACATGCTCAGAAGAGGTTGGCCA
CCTCACAGAACACTCCGTGGTCATCGGAACAAAGTCACATGTTTGCTATATCCTCATCAGGTCTCAGCT
CGGTATGATCAAAGATACCTGATATCTGGAGGTGTGGATTTTTCAGTCATAATTTGGGACATATTTTCT
GGAGAAATGAAACATATCTTCTGTGTTCATGGTGGTGAGATTACTCAACTTCTAGTTCCACCTGAAAAC
TGTAGTGCAAGAGTACAGCACTGCATCTGCTCTGTAGCCAGTGACCACTCAGTAGGACTTCTAAGTTT
GCGAGAGAAAAAATGCATAATGTTGGCATCTCGTCACCTTTTTCCTATTCAAGTAATCAAATGGAGGC
CTTCTGATGATTACCTGGTGGTGGGGTGTTCAGATGGTTCTGTGTACGTCTGGCAAATGGATACTGGTG
CATTGGATCGTTGTGTGATGGGGATAACAGCAGTTGAGATTCTAAACGCTTGTGATGAAGCTGTTCCT
GCTGCTGTTGATTCACTTAGTCATCCAGCAGTCAACCTAAAACAAGCTATGACGAGACGTAGTCTTGCT
GCTCTTAAAAATATGGCCCATCATAAGCTACAAACCCTTGCAACTAACCTCTTGGCTTCTGAGGCATCT
GACAAGGGAAATTTACCTAAATATTCTCATAACTCCCTGATGGTTCAAGCAATAAAGACAAACCTAAC
AGACCCGGACATACATGTGCTATTCTTTGATGTGGAAGCGTTGATTATTCAACTCCTGACTGAAGAAG
CCTCTAGGCCGAATACTGCTCTTATTTCCCCAGAGAATTTGCAAAAAGCATCTGGCAGTTCAGACAAA
GGGGGCTCTTTTTTAACTGGAAAACGAGCAGCAGTTCTCTTCCAACAAGTGAAAGAAACGATCAAAGA
GAACATCAAGGAACACCTCCTTGATGATGAAGAGGAGGATGAGGAGATAATGAGGCAGAGAAGGGA
AGAAAGTGATCCTGAATATCGGTCCAGCAAATCAAAGCCATTGACCCTATTAGAATATAATTTAACTA
TGGACACTGCAAAGCTGTTTATGTCCTGCCTTCACGCCTGGGGTTTGAATGAAGTACTGGATGAAGTTT
GCCTGGATCGCCTTGGAATGCTGAAACCCCACTGCACCGTATCGTTTGGCCTCTTGTCAAGAGGAGGC
CATATGTCACTGATGCTGCCGGGTTATAATCAGCCTGCTTGTAAACTGTCACATGGGAAAACAGAAGT
AGGAAGGAAGCTGCCAGCGTCTGAGGGAGTAGGAAAGGGAACTTACGGAGTGTCCCGTGCCGTCACC
ACACAGCATCTCCTGTCTATCATTTCTTTGGCAAATACTTTAATGAGTATGACCAATGCAACTTTTATT

GGTGATCATATGAAGAAGGGTCCTACCAGGCCACCTAGACCAAGCACCCCAGACCTTTCTAAGGCAAG
GGGTTCCCCTCCAACTTCCAGTAATATTGTGCAAGGACAGATTAAACAAGTTGCTGCACCTGTCGTTTC
CGCTCGGTCTGATGCTGATCACTCTGGCTCTGACCCTCCTTCTGCTCCTGCTTTACATACCTGTTTCTTA
GTAAATGAAGGTTGGAGTCAGTTAGCTGCTATGCACTGTGTTATGCTGCCAGACCTACTGGGATTGGA
TAAATTTAGGCCTCCCCTTCTGGAGATGCTGGCCCGAAGAT_GCAAGATCGATGCTTGGAGGTGAGAG
AAGCCGCGCAGGCCCTGCTTCTGGCGGAACTGAGAAGAATTGAGCAGGCAGGCAGGAAGGAAGCCAT
TGATGCCTGGGCTCCTTACTTACCTCAGTACATAGACCACGTCATATCACCTGGAGTCACATCAGAAGC
CGCGCAGACTATCACCACGGCTCCTGATGCCTCAGGGCCTGAAGCAAAAGTCCAGGAGGAAGAGCAT
GACCTTGTTGACGATGACATCACCACTGGTTGCTTATCAAGTGTCCCACAAATGAAAAAAATTTCTAC
ATCTTACGAGGAAAGACGGAAGCAAGCTACCGCTATTGTTTTACTTGGAGTAATAGGAGCTGAATTTG
GTGCTGAAATTGAACCTCCTAAACTATTGACCAGACCTCGAAGCTCTAGCCAAATTCCTGAGGGATTC
GGGTTGACTAGTGGTGGATCCAACTACTCGCTGGCCAGACATACTTGCAAGGCACTGACGTTTCTTCTG
CTACAGCCTCCAAGCCCCAAACTTCCTCCACACAGCACTATCCGAAGAACAGCCATTGATCTGATTGG
ACGTGGGTTCACTGTTTGGGAGCCTTACATGGATGTGTCCGCTGTTCTGATGGGGCTTCTCGAACTTTG
TGCCGATGCCGAGAAACAACTTGCCAACATCACAATGGGGTTGCCTCTGAGCCCAGCAGCTGACTCGG
CCCGCTCTGCGAGGCATGCCCTCTCGCTCATTGCCACCGCCAGACCACCCGCCTTCATCACCACCATAG
CCAAAGAGGTACACAGACATACGGCTCTTGCAGCAAATACCCAATCACAGCAGAATATGCACACAAC
AACTCTTGCACGAGCTAAAGGGGAAATTTTGAGAGTCATTGAAATTCTTATTGAAAAGATGCCCACAG
ATGTTGTGGATCTTCTCGTGGAGGTTATGGACATCATTATGTACTGCCTTGAAGGATCTTTAGTTAAAA
AGAAAGGTCTTCAAGAATGTTTCCCAGCCATCTGCAGGTTCTACATGGTCAGCTATTATGAGCGGAAT
CACAGAATAGCAGTTGGAGCTCGCCATGGTTCAGTGGCCCTGTACGACATCCGGACTGGAAAATGTCA
GACAATCCATGGACACAAGGGACCAATCACTGCAGTGGCTTTTGCTCCTGATGGAAGATATCTTGCCA
CCTACTCAAACACTGACAGCCACATTTCTTTTTGGCAGATGAACACGTCACTGCTGGGAAGCATCGGC
ATGCTGAACTCGGCACCTCAGCTGCGCTGCATTAAAACCTACCAGGTGCCCCCTGTGCAGCCCGCGTC
CCCCGGCTCCCACAATGCCCTCAAGCTGGCCCGGCTCATCTGGACTTCCAACCGCAACGTCATCCTCAT
GGCCCATGACGGGAAGGAGCACCGCTTATGGTCTAATGCTGCTGCCTGCCGCCGTGACTGCGTTTTAG
TTCTCTAAATTATCCAAGCCGATGTTGCTCTGTCCTTCCTCACACCAGATTGTTCCCAGGGGCCTGCCC
ACCCCAGTGCCATCCAGTGGCACGGCCGGGTCTTGTCACTTGTGCATGCTTCTCAGGGGCAGAACCCG
CTCGTGCCATCTGTCGATTCAGAGGCACGCACACATGCTCTGCAGGATGTGGCCATCCTGTCACCAGG
TAGTTTTTCCCTGCCAGAGATGGCAGGGGAAAGCCAGTGGTTCCTGGGAACGCTCTTGTTGCTTGGTGC
AACAGAAGCACAAAAATCAATAAACACTGTGATTGCACTCCCAGCCCAGATCAGCATTTTTAGCCATC
TCAACCGCACCTCTGAAGTGCTGCTTGAATCTGGCCGTTCTGACACTGGGTGTTGAGGTCATGTGGCAG
TCCTCACGATTGAAATTATTGCCCTTTGAATTACCTAAAATATTGTTTACCAAAAAGATTCTGTGTTTA
CATGTTCTTTTCTTCTGGTGATTGCTAGGAAATTACTCAATCCAATTTCCTGGGTTTAGCTACCACCTTC
TTTATCAACAGCTAACCAATTTGAGATTCATGATTAATTATGTACACAAAAATATTTTCCTTGTAGAAA
ATAGTTTTCCAGACACTTAGCCTTCTGAAGTGCTCATCTCTTGCTTAAATGATAGTCTATTTAGTAGTTT
AAAATAAAAATAATCACTCCCTATGATCTAGTTAATGTAATTATCCAGTCACTGTAACTAATCTCTTGA
AGGGACTTTTGTGATAAAGAGCCAAGTTCAGAAGCATAGCGTGTACCGAAAATGAAGACTCTCCTATC
TACTGCTACAGATCCTGTTGAAGAAAGAGCTTACCCCTTTCCCCCGGAAAACTTGCAATCCTGTAGTTC
CCATATGTGTGGCATATGTCAGTTATGAAATTATTATAATTCAGCCTTCAAACTACTACTGATCAACTA
AAGGAAAGCTAATAAGGTATTTCTTTTAAACAAGTATTCTGGAAAAGTAATAACTATGGCACAAATGT
TCAGGAGTGTATATAAAAATATGTGGGCTCTCGATTTATTTGAGCAAACATTGCAATGAGGGTGTTCA
TTATGCTTCTAAGTAAGCTTTAACTAGACCACTTTCTTCTTATGTCAGATTGTGCTGGTTCTAGCTCATA
ATAATGAAAAAAATAGAATTATATTTTGTATGAATTGTTTCTAACAAACCTTGATCTGTATATACTTGT
GAGGGGTTGTTTGGTTGCCTAATTGTTGATTGGTTGGTTAGTTGGGCAAAATCATTTATTACATGCAAA
AATAGAAAATTATTTGTTAAATTCAGAGAAAATATGTGAAGTTTACAAAGGAACCAAAATGATAATGG
GAGAATTTAGTCTTAAAAGTTTAGGTTGTCAGAGTTTATCTGATATACAAAAAAGTAGGATCAAAGTC
TAGGAACAAACATTGGTTATTTCACTGAAACAGATTTTTAAAGAGCATATTAAGAAATTAAGACCATT
AGATCATCTCTTTCATAATGATATGATGTTATTTCCATTGACGGCAAATCTATACTGTTCCTTGGTTTTA
GAGTTGGGTATTCTTTCAAAAGTGAATTTAGGCACATGCATGTACATTGTATTTATAAAGATTATATAT
GTAGTTTCCAAGGAAGAAACAAAGACCTATACAGTTTAGACTTTATTTGTTTGGTGAACGTGCTGTTGT
GTTTGGCATAGCTAGAGAAATGTAAGGTAAAAGGCTCACTTTGTTTTCCAGTGGGTCCAGAGTCTTCTC
TATACCCAACATATGTTTACTTTATGAACTTAAATGGAAATTAATTTATTGTAGACTACATCTCTATTTG
GAATGATGAGGTAAATGTATACCTTAGCATTTTCTTTAACAAAAATGTTCTGTGTGTCAGGAAAGAGG
TGTGAGAGTGGTAGGCAGCCTGCCGCAAACACACGGATGGCTCCCCCCGCCACGAAGGTTGAGAACA
AAGCCAGGGGGCCCAGAGGAGCAATAGCCCTGGGCCACAAGCGTGGGTAGGCCTTTCACGTATTCCG

TCTGCACAGGCAACCAAGGCCAGTAGAAAGCTATGGCTGCAAAACCCTGGGGTGGACGATGTTTGAT
GATTAGACGGTCATCTCTAACTACTAAACTAATATCTGCATTCTTGTTAACATAAATCTCTCCTTGGCT
ATTTTAATAGCCACAGTCCCAAACTTCATCCCAGCTTAGATTTCTCAGCACTGAGGTAAACTCTGCATT
GTCATCCCAGCGTACACCTTTAATGTTTTATTGATTTCACTGGCACTGTATTTGGACCTGTCCTTGTATA
TGTAGAGACATATGTGGCTTCATTGGTAATTTACAAGCAAAAGATGACATGACGTGACACCTGTATGA
AAATGTGATGATTGAACTCCCTGTGTACAGCGAGTGAATTAAAATGTCTGCCTCTCCAGACATTTCTTA
ATGATTCCACTTAATAGACTCTATGTGTGCTGAATGTTCCTGTGTACATATGTGTGTTAAATAAAACAA
TTGTATTGAA

J) Amino acid sequence: WDR7 protein truncation (1023aa)
MAGNSLVLPIVLWGRKAPTHCISAVLLTDDGATIVTGCHDGQICLWDLSVELQINPRALL
FGHTASITCLSKACASSDKQYIVSASESGEMCLWDVSDGRCIEFTKLACTHTGIQFYQFS
VGNQREGRLLCHGHYPEILVVDATSLEVLYSLVSKISPDWISSMSIIRSHRTQEDTVVAL
SVTGILKVWIVTSEISDMQDTEPIFEEESKPIYCQNCQSISFCAFTQRSLLVVCSKYWRV
FDAGDYSLLCSGPSENGQTWTGGDFVSSDKVIIWTENGQSYIYKLPASCLPASDSFRSDV
GKAVENLIPPVQHILLDRKDKELLICPPVTRFFYGCREYFHKLLIQGDSSGRLNIWNISD
TADKQGSEEGLAMTTSISLQEAFDKLNPCPAGIIDQLSVIPNSNEPLKVTASVYIPAHGR
LVCGREDGSIVIVPATQTAIVQLLQGEHMLRRGWPPHRTLRGHRNKVTCLLYPHQVSARY
DQRYLISGGVDFSVIIWDIFSGEMKHIFCVHGGEITQLLVPPENCSARVQHCICSVASDH
SVGLLSLREKKCIMLASRHLFPIQVIKWRPSDDYLVVGCSDGSVYVWQMDTGALDRCVMG
ITAVEILNACDEAVPAAVDSLSHPAVNLKQAMTRRSLAALKNMAHHKLQTLATNLLASEA
SDKGNLPKYSHNSLMVQAIKTNLTDPDIHVLFFDVEALIIQLLTEEASRPNTALISPENL
QKASGSSDKGGSFLTGKRAAVLFQQVKETIKENIKEHLLDDEEEDEEIMRQRREESDPEY
RSSKSKPLTLLEYNLTMDTAKLFMSCLHAWGLNEVLDEVCLDRLGMLKPHCTVSFGLLSR
GGHMSLMLPGYNQPACKLSHGKTEVGRKLPASEGVGKGTYGVSRAVTTQHLLSIISLANT
LMSMTNATFIGDHMKKGPTRPPRPSTPDLSKARGSPPTSSNIVQGQIKQVAAPVVSARSD
ADHSGSDPPSAPALHTCFLVNEGWSQLAAMHCVMLPDLLGLDKFRPPLLEMLARRCKIDA
WR*

II. *WDR7* gene sequences of wild-type and WDR7 knock-out A549 cells obtained by Sanger sequencing.

A, C. Wild-type A549 cells. **B, D.** Negative CT control cells.
Results of Sanger sequencing of wild-type A549 cells and negative CT control cells.
The sgRNA binding regions of exon 2 and exon 17 are highlighted in yellow.

A) Nucleotide sequence of *WDR7* gene, exon 2 region, in wild-type A549 cellsAGCGCCCACACATTGCATCTCAGTAGGTACTTTTAACAGATGATGGGGCCACGATCGTAACAGGA
TGTCACGACGGACAAATATGTCTCTGGGATCTTTCAGTAGAACTGCAAGTGAGTATGTGAAATGCCTA
TTATACATTTTAGATATAAAAATCTACTTATTAGAAGATAATATAGTCCCCGAATGATGCTTTTGGGCC
CACAGTAGATTTTCTGAAAAATGTGTTTTGACTAAAACTAGTAGTATACAGATTCATTATTTACACTAT
TCATCAGAGTTGACTTCTGAGGGCTTTTGGCCATTTTCAATTGTAATATCTACCCTAAGTGACTTACTGT
TTTGTTACATTTAAGATTTTTCAAATGATGATGACCCATACTGAAGAAGGTTTTCCAAAGAGGTTTTAG
AAAATTTAAAACACTTAAAACCTTCTATGGTAAATGCTTTGAAGAGGGCAAAACTTTTTACAAAGTAT
AGATTTTGTTGTATTGATTAATGACTGTGCCTTTATTGTCATTCCTTACTACTATGGATTGCTCAGTGTG
CTCTGCTTCCAGGA

B) Nucleotide sequence of *WDR7* gene, exon 2 region, in negative CT control cells
CGGTACTTTTAACAGATGATGGGGCCACGATCGTAACAGGATGTCACGACGGACAAATATGTCTCTGG
GATCTTTCAGTAGAACTGCAAGTGAGTATGTGAAATGCCTATTATACATTTTAGATATAAAAATCTACT
TATTAGAAGATAATATAGTCCCCGAATGATGCTTTTGGGCCCACAGTAGATTTTCTGAAAAATGTGTTT
TGACTAAAACTAGTAGTATACAGATTCATTATTTACACTATTCATCAGAGTTGACTTCTGAGGGCTTTT
GGCCATTTTCAATTGTAATATCTACCCTAAGTGACTTACTGTTTTGTTACATTTAAGATTTTTCAAATGA

TGATGACCCATACTGAAGAAGGTTTTCCAAAGAGGTTTTAGAAAATTTAAAACACTTAAAACCTTCTA
TGGTAAATGCTTTGAAGAGGGCAAAACTTTTTACAAAGTATAGATTTTGTTGTATTGATTAATGACTGT
GCCTTTATTGTCATTCCTTACTACTATGGATTGCTCAGTGTGCTCTGCTTCCA

C) Nucleotide sequence of *WDR7* gene, exon 17 region, in wild-type A549 cells
ACACAGTGGTAACACACAGATGGCCATGCCTTAAGTGACATGAACCTATTTTTGGATATCTGTGTGAA
TGCCCTTTTAACTGACAGTGGTTTCAAATCTATAGCTCTAGTATGTGTTTATTTCTGTGTTTTGCCATTC
TTTAATATATATTTTATTTTAGAGGGACTGTTAAGAAATGTGTTTTTCTTGTGAATTTGGATATACTTCC
AGTGGAAATTTATTTTTATTTCTTTTAAGATGTTGAGAATTCTTATAGGCACCAATTACATGATTTAGG
GATGAAATACGTTTAGTATAATTCTTTGCACAAAGTGTCTACTATTAAATTGAAATCATTTGTAATATA
ACTTAGCAGATCTGCCTTTTTTTGTTTTTGGTATACTTACATAAAGAACTGTTGACTTAGGGAATGCCA
AAATGGTTAAAGAATTTGTAATATATTACATTTTTGACAGGTTGGAGTCAGTTAGCTGCTATGCACTGT
GTTATGCTGCCAGACCTACTGGGATTGGATAAATTTAGGCCTCCCCTTCTGGAGATGCTGGCCCGAAG
ATGGCAAGATCGATGCTTGGAGGTAATGCTAAAGTGATAAGGATAGAAAACAAAAATATTAAAAAGG
CATTGGTCAAATGTAAACTTGAAACAAAAATGATTACTGTTTATCTGATTTATCATCTGTTCATTATGT
GATAGAAAATGTGGATTCATGATTTATGTTTTCTTCTTTGTCAGCTTTTATACCAACCAGTAATGCTCCA
GCTGGTTTATTCGTTTACTTTTAAAGAATTCAGTGACTGTTTGATGAAGTCTTATATACTTTATGGAACT
ATTTCTTAAGTTTGGTTTCTGTACGGATCACACACACTCATGC

D) Nucleotide sequence of *WDR7* gene, exon 17 region, in negative CT control cells
TGACAGTGGTTTCAAATCTATAGCTCTAGTATGTGTTTATTTCTGTGTTTTGCCATTCTTTAATATATAT
TTTATTTTAGAGGGACTGTTAAGAAATGTGTTTTTCTTGTGAATTTGGATATACTTCCAGTGGAAATTT
ATTTTTATTTCTTTTAAGATGTTGAGAATTCTTATAGGCACCAATTACATGATTTAGGGATGAAATACG
TTTAGTATAATTCTTTGCACAAAGTGTCTACTATTAAATTGAAATCATTTGTAATATAACTTAGCAGAT
CTGCCTTTTTTTGTTTTTGGTATACTTACATAAAGAACTGTTGACTTAGGGAATGCCAAAATGGTTAAA
GAATTTGTAATATATTACATTTTTGACAGGTTGGAGTCAGTTAGCTGCTATGCACTGTGTTATGCTGCC
AGACCTACTGGGATTGGATAAATTTAGGCCTCCCCTTCTGGAGATGCTGGCCCGAAGATGGCAAGATC
GATGCTTGGAGGTAATGCTAAAGTGATAAGGATAGAAAACAAAAATATTAAAAAGGCATTGGTCAAA
TGTAAACTTGAAACAAAAATGATTACTGTTTATCTGATTTATCATCTGTTCATTATGTGATAGAAAATG
TGGATTCATGATTTATGTTTTCTTCTTTGTCAGCTTTTATACCAACCAGTAATGCTCCAGCTGGTTTATT
CGTTTACTTTTAAAGAATTCAGTGACTGTTTGATGAAGTCTTATATACTTTATGGAACTATTTCTTAAGT
TGGTTCT
