# Supplementary material for: Identification of Host Factors for Rift Valley Fever Phlebovirus
Source: Viruses. 2023 Nov 13;15(11):2251. doi: 10.3390/v15112251 (PMC10675714; doi:10.3390/v15112251)
Supplement: Supplementary file 1 [file viruses-15-02251-s001.zip › Supplementary_figures corrected FINAL JAR.pdf]

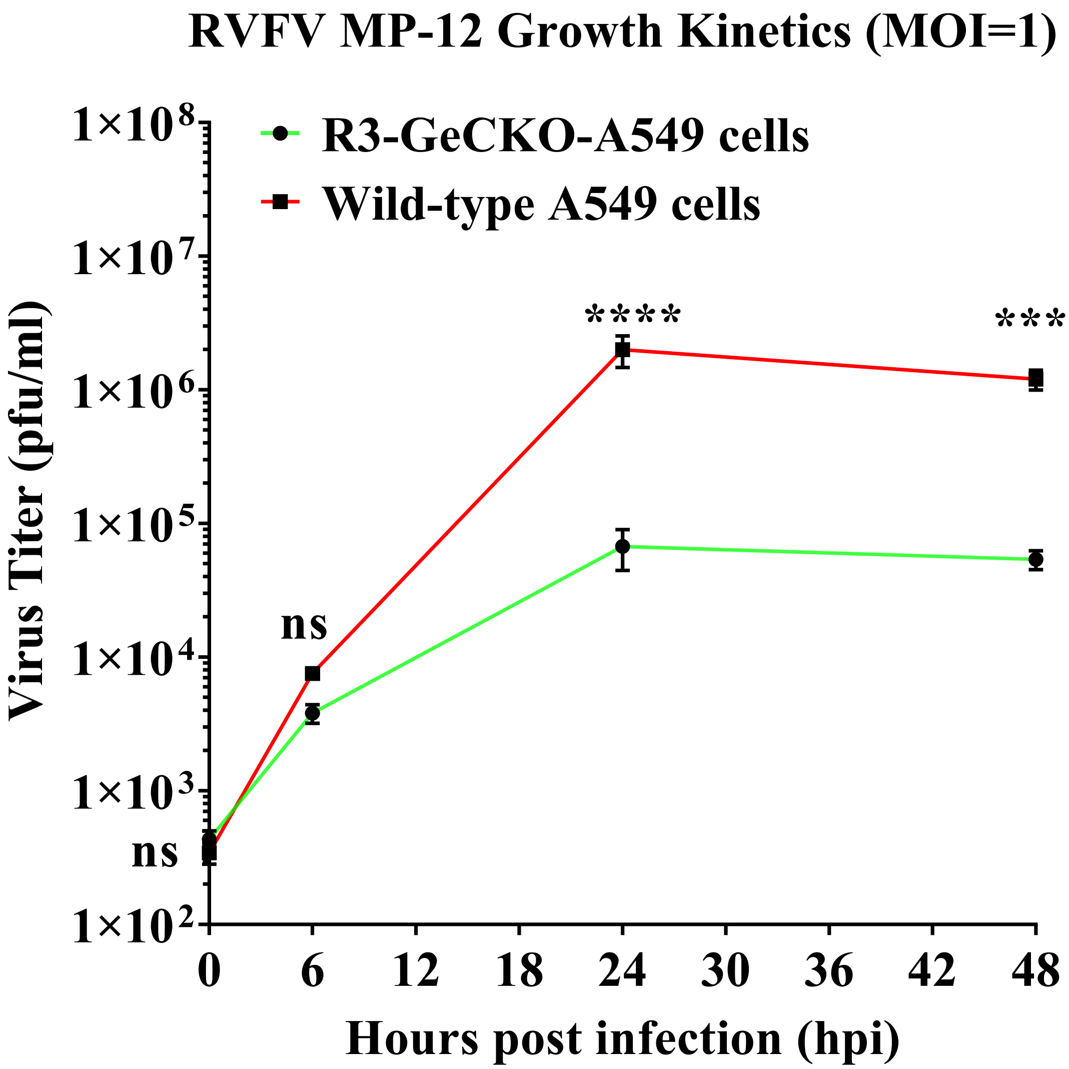

Supplementary Figure S1:

RVFV growth kinetics on wild-type and round 3 GeCKOA549 cells. The figure illustrates MP-12 virus titers at various time points (hpi) in wild-type and round 3 (R3) GeCKO-A549 cells. Statistical analysis was performed on one independent experiment with three replicates, using a 2-way ANOVA Sidak's multiple comparisons test (**** p-value <0.0001; ns=non-significant).

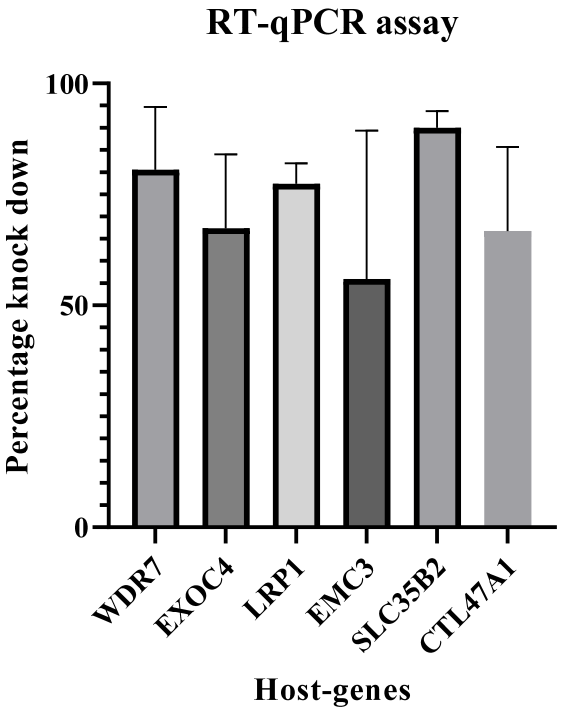

Supplementary Figure S2:

Confirmation of *WDR7* gene knock-down. A549 cells were transfected with 50 nM of gene-specific siRNAs. Forty-eight hours post-transfection, the total cellular RNA was extracted, and two-step RT-qPCR assay was performed to determine the percentage of gene knock-down. *WDR7*, *EXOC4, LRP1, EMC3, SLC35B2*, and *CTL47A1* gene-specific siRNAs were transfected into wild-type A549 cells. Each bar represents the average percentage of gene knock-down with standard deviation.

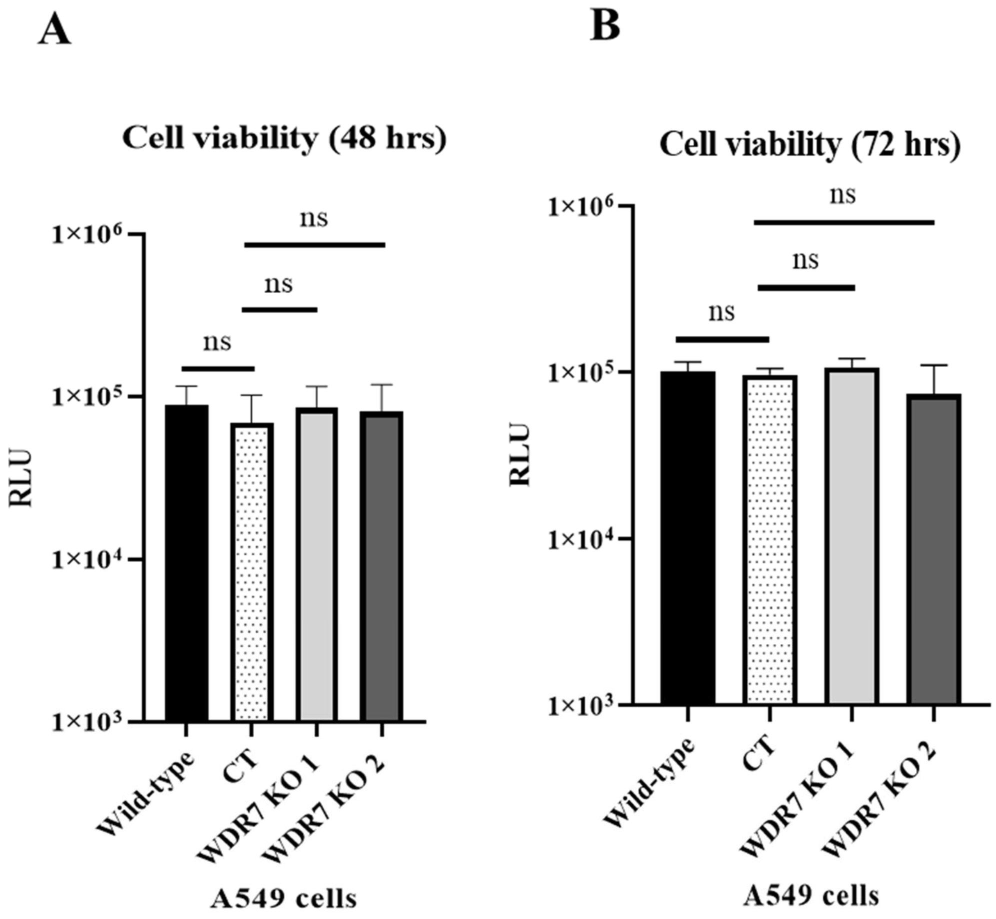

A
Cell viability (48 hrs)
RLU
1×10⁶
1×10⁵
1×10⁴
1×10³
ns
ns
ns
Wild-type
CT
WDR7 KO 1
WDR7 KO 2
A549 cells
B
Cell viability (72 hrs)
RLU
1×10⁶
1×10⁵
1×10⁴
1×10³
ns
ns
ns
Wild-type
CT
WDR7 KO 1
WDR7 KO 2
A549 cells

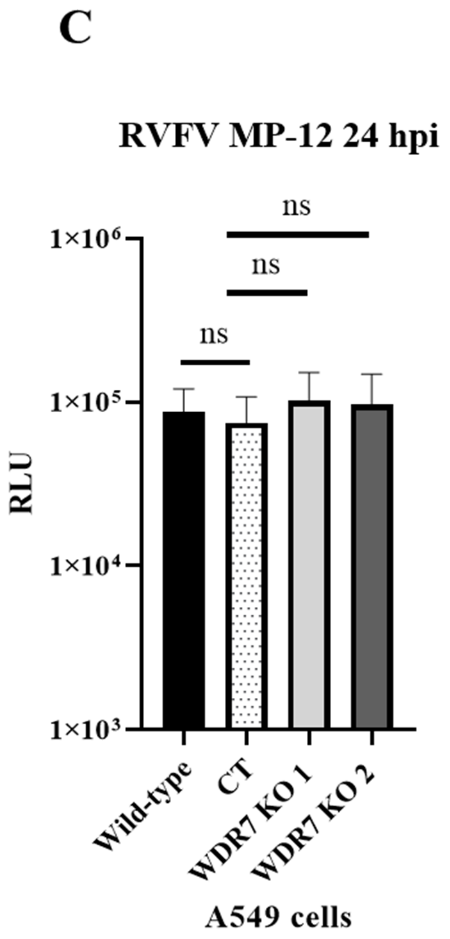
C
RVFV MP-12 24 hpi
ns
ns
ns
1×10⁶
1×10⁵
1×10⁴
1×10³
RLU
Wild-type
CT
WDR7 KO 1
WDR7 KO 2
A549 cells

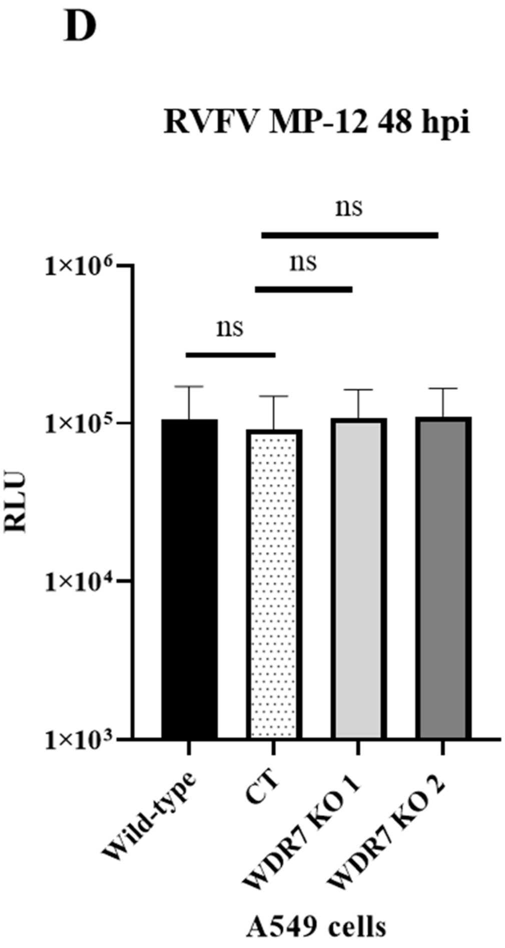
D
RVFV MP-12 48 hpi
ns
ns
ns
1×10⁶
1×10⁵
1×10⁴
1×10³
RLU
Wild-type
CT
WDR7 KO 1
WDR7 KO 2
A549 cells

E

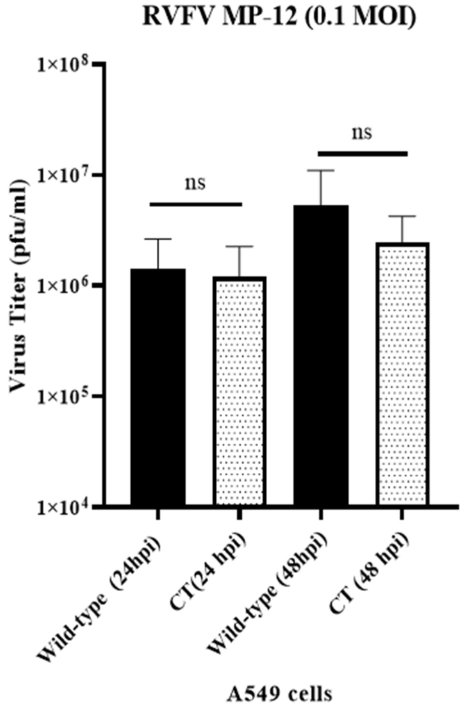

Supplementary Figure S3:

Cell viability and virus titers of wild-type, CT, and *WDR7* gene knock-out A549 cell lines.

A, B. Uninfected wild-type, CT, and *WDR*7 gene knock-out A549 cell lines 1 and 2: The wild-type, CT and *WDR7* gene knock-out (KO) A549 cells were seeded, and at different time points (48 and 72 hrs) post-seeding, the cell viability was measured using the ADP cell-glow assay. **C, D**. Wild-type, CT, and *WDR*7 gene knock-out A549 cell lines 1 and 2 infected with MP-12: The cell viability of wild-type, CT and *WDR7* gene KO cells were measured after RVFV MP-12 infection at 24 and 48 hpi. Each bar represents the average Relative Light Unit (RLU; A, B, C, D) with standard deviation. **E**. Virus titers of wild-type and control CT A549 cells infected with MP-12 at 24 and 48 hpi. Each bar represents the average virus titer (pfu/ml) with standard deviation determined by plaque assay. Statistical analysis was done on two independent experiments with four technical replicates for each, using Mann-Whitney U independent Student's t-test (ns=non-significant).

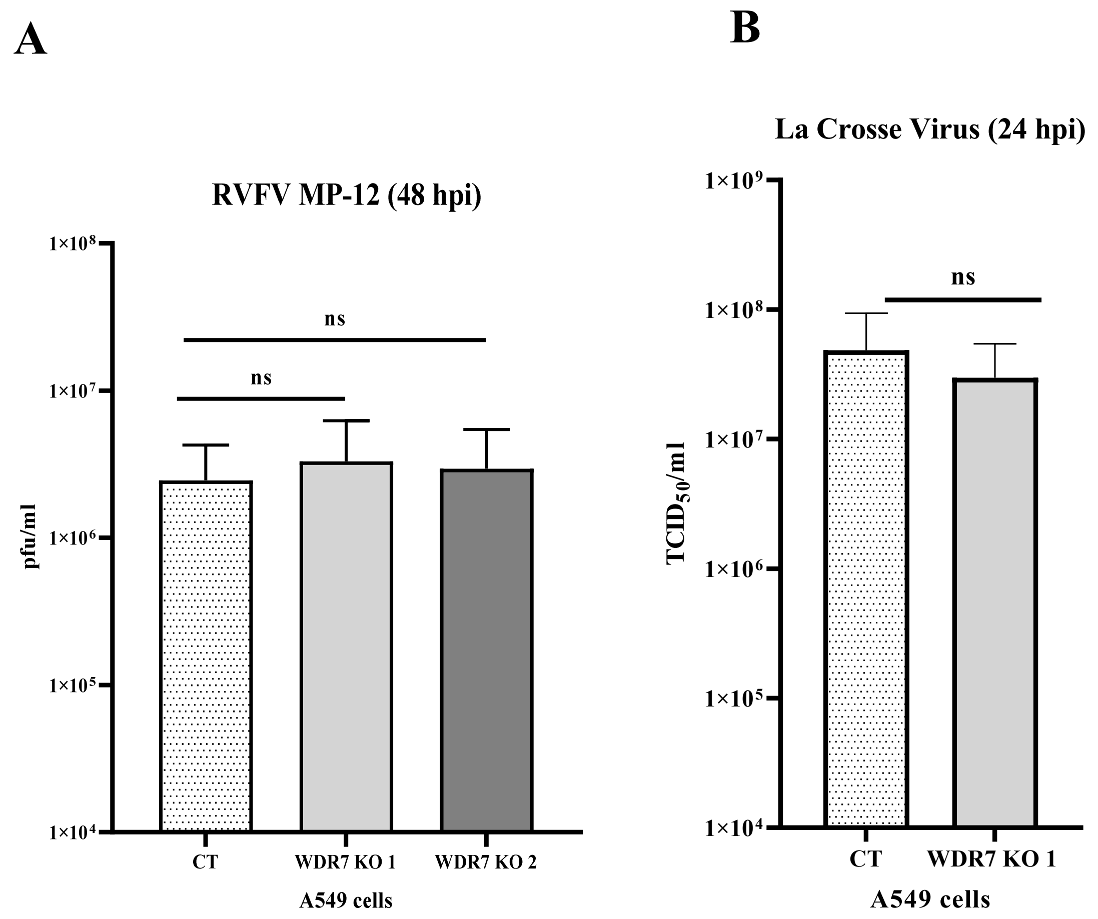

Supplementary Figure S4:

Effect of *WDR7* gene KO cell lines 1 and 2 on virus replication of RVFV and LACV at 48 or 24 hpi, respectively. CT and *WDR7* gene knock-out cells were infected with (**A**) RVFV MP-12 or (**B**) LACV virus at 0.1 MOI. At 24- or 48-hours post-infection (hpi), the supernatant was collected and titered by plaque assay (RVFV) or $TCID_{50}$-CPE assay (LACV). CT= non knock-out A549 cells and *WDR7* gene knock-out cell lines 1 and 2. Three independent experiments with three technical replicates each, were performed for RVFV MP-12, and two independent experiments with eight technical replicates each, were performed for LACV. Statistical analysis was done using Mann-Whitney U independent Student's t-test (ns=non-significant).
