# Supplementary material for: Identification of Host Factors for Rift Valley Fever Phlebovirus
Source: Viruses. 2023 Nov 13;15(11):2251. doi: 10.3390/v15112251 (PMC10675714; doi:10.3390/v15112251)
Supplement: Supplementary file 1 [file viruses-15-02251-s001.zip › Supplementary_Tables corrected FINAL JAR.pdf]

Supplementary Table S1:

Location of the *indels* in the *WDR7* gene of knock-out A549 cells. Represented are the predominant *indels* in the *WDR7* gene of the WDR7 knock-out cell lines 1 and 2. The sgRNA binding sites on the *WDR7* gene are highlighted in yellow and the *indels* and the corresponding amino acid changes are highlighted in red. Predicted full nucleotide and amino acid sequences are included in the Supplementary File S2-I.

**Indels**	**Nucleotide sequence-*WDR7* gene (5'-3')**	**Amino acid sequence**
Predicted nucleotide sequence-WDR7 gene - Exon-2	**ATG…..ACGATCGTAACAGGATGTCACGAC…**	M…TIVTGCHD…
WDR7 KO cell population 1- Exon-2- **'A' insertion**	**ATG…..ACGAATCGTAACAGGATGTCACGAC..**	M…TNRNRMSR… (frame shift mutation-early termination)
WDR7 KO cell population 1-Exon-2- **'T' deletion**	**ATG…..ACGA_CGTAACAGGATGTCACGAC..**	M…TT* (Stop Codon)
Predicted nucleotide sequence -WDR7 gene - Exon-17	**ATG………CGAAGATGGCAAGATCGATGCTTGG….**	M…RRWQRCL…
WDR7 KO cell population 2- Exon-17- **'T' insertion**	**ATG………CGAAGATTGGCAAGATCGATGCTTGG….**	M…RRLARSML… (frame shift mutation-truncation)
WDR7 KO cell population 2- Exon-17- **'G' deletion**	**ATG………CGAAGAT_GCAAGATCGATGCTTGG….**	M…RRCKIDAW… (frame shift mutation-truncation)

Supplementary Table S2.

Primers used for RT-qPCR, NGS and Sanger sequencing.

Gene	Primer	Sequence
WDR7	WDR7-1 Forward	TTTATGCCACGGACATTACCC
	WDR7-1 Reverse	GGAGCTAATCCAGTCTGGTGATA
LRP1	LRP1-1 Forward	AGCCAGCTATGCACCAACAC
	LRP1-1 Reverse	CCTTGCAGGAGCGGTTATC
SLC35B2	SLC35B2-1 Forward	AGGTGATCCCTGTCATGCTGA
	SLC35B2-1 reverse	CGCTGGATAGCAGAAACATGC
EMC3	EMC3-1 Forward	GTGGTCCTACCCATCGTTATC
	EMC3-1 Reverse	CAGATACTTGTTCCTGGGTGAG
EXOC4	EXOC4-1 Forward	ACAGGTACGTTAATAGTTAATAGCGT
	EXOC4-1 Reverse	TCGTCAATTCTGTGTAGTGCTG
GAPDH	GAPDH Forward	TGTAGTTGAGGTCAATGAAGGG
	GAPDH Reverse	ACATCGCTCAGACACCATG
CT47A1	CT47A1-1 Forward	CGTCTGAGACAGACTCTTATTCC
	CT47A1-1 Reverse	TGACCACTGAGGTGGCTA
WDR7 Exon 2	Wsg1-indel-R1-Forward	CTTTCCCTACACGACGCTCTTCCGATCTcCACAAACACAATGGCAGGAAACAG
	Wsg1-indel-R1-Reverse	GACTGGAGTTCAGACGTGTGCTCTTCCGATCT-GGCCCAAAAGCATCATTTGG
WDR7 Exon 17	Wsg5-indel-R1-Forward	CTTTCCCTACACGACGCTCTTCCGATCTtttGACAGGTTGGAGTCAGTTAGCTGC
	Wsg5-indel-R1-Reverse	GACTGGAGTTCAGACGTGTGCTCTTCCGATCT-CAAGTTTACATTTGACCAATGCC
NGS- WDR7 Exon 2	NGS-indel-R2-Forward-1	AATGATACGGCGACCACCGAGATCTACACTATAGCCTACACTCTTTCCCTACACGACGCTCTTCC
	NGS-indel-R2-Reverse-1	CAAGCAGAAGACGGCATACGAGATCGAGTAATGTGACTGGAGTTCAGACGTGTGCTCTTC
NGS-WDR7 Exon 17	NGS-indel-R2-Forward-2	AATGATACGGCGACCACCGAGATCTACACATAGAGGCACACTCTTTCCCTACACGACGCTCTTCC
	NGS-indel-R2-Reverse-2	CAAGCAGAAGACGGCATACGAGATTCTCCGGAGTGACTGGAGTTCAGACGTGTGCTCTTC
Sanger - WDR7 Exon 2	sWsg1 Forward	TCCCAGCAGGATCTACGCAC
	sWsg1 Reverse	GTCCTCTTGTGTTCGGTGGG
Sanger-WDR7 Exon 17	sWsg5 Forward	GCCACCTAGACCAAGCACC
	sWsg5 Reverse	CGGATAGTGCTGTGTGGAGG

Supplementary Table S3:

Primer and probes for the detection of viral RNA by RT-qPCR.

Gene	Primer/Probe	Sequence	Ref.
LACV gene L	RT-qPCR LAC fwd	AGGAAAACTCCTGAGAATATAACTA	(none)
	RT-qPCR LAC rev	GGTATACAAACTGGTGGCGAT	
	Rt-qPCR LAC probe	6-FAM-CTTAAATTTGAAAATATGTCTAAAATCCAAACATACCCAGGC-BHQ-1	
RVFV gene L	RVFL-2912fwdGG	TGAAAATTCCTGAGACACATGG	Bird et al., JCM, 2007, 45:11(3506-13)
	RVFL-2981revAC	ACTTCCTTGCATCATCTGATG	
	RVFL-probe-2950	6-FAM-CAATGTAAGGGGCCTGTGTGGACTTGTG-BHQ1	
PGK1 gene	PGK1 fwd	GCCACTTGCTGTGCCAAATG	OriGene, Rockville, MD, USA
	PGK1 rev	CCCAGGAAGGACTTTACCTT	

Fwd=forward; rev=reverse; OriGene: PGK1 primer sequence was obtained from OriGene
